# A scoping review of scoping reviews: advancing the approach and enhancing the consistency

**DOI:** 10.1002/jrsm.1123

**Published:** 2014-07-24

**Authors:** Mai T Pham, Andrijana Rajić, Judy D Greig, Jan M Sargeant, Andrew Papadopoulos, Scott A McEwen

**Affiliations:** a Department of Population Medicine, Ontario Veterinary College, University of GuelphGuelph, Ontario, N1G 2W1, Canada; b Division of Public Health Risk Sciences, Laboratory for Foodborne Zoonoses, Public Health Agency of Canada160 Research Lane, Suite 206, Guelph, Ontario, N1G 5B2, Canada; c Food Safety and Quality Unit, Food and Agriculture Organization of the United NationsViale delle Terme di Caracalla, 00153, Rome, Italy; d Centre for Public Health and Zoonoses, Ontario Veterinary College, University of GuelphGuelph, Ontario, N1G 2W1, Canada

**Keywords:** scoping review, literature review, knowledge synthesis, methodology

## Abstract

**Background:**

The scoping review has become an increasingly popular approach for synthesizing research evidence. It is a relatively new approach for which a universal study definition or definitive procedure has not been established. The purpose of this scoping review was to provide an overview of scoping reviews in the literature.

**Methods:**

A scoping review was conducted using the Arksey and O'Malley framework. A search was conducted in four bibliographic databases and the gray literature to identify scoping review studies. Review selection and characterization were performed by two independent reviewers using pretested forms.

**Results:**

The search identified 344 scoping reviews published from 1999 to October 2012. The reviews varied in terms of purpose, methodology, and detail of reporting. Nearly three-quarter of reviews (74.1%) addressed a health topic. Study completion times varied from 2 weeks to 20 months, and 51% utilized a published methodological framework. Quality assessment of included studies was infrequently performed (22.38%).

**Conclusions:**

Scoping reviews are a relatively new but increasingly common approach for mapping broad topics. Because of variability in their conduct, there is a need for their methodological standardization to ensure the utility and strength of evidence. © 2014 The Authors. *Research Synthesis Methods* published by John Wiley & Sons, Ltd.

## 1. Background

The scoping review has become an increasingly popular approach for synthesizing research evidence (Davis *et al.*, 2009; Levac *et al.*, 2010; Daudt *et al.*, 2013). It aims to map the existing literature in a field of interest in terms of the volume, nature, and characteristics of the primary research (Arksey and O'Malley, 2005). A scoping review of a body of literature can be of particular use when the topic has not yet been extensively reviewed or is of a complex or heterogeneous nature (Mays *et al.*, 2001). They are commonly undertaken to examine the extent, range, and nature of research activity in a topic area; determine the value and potential scope and cost of undertaking a full systematic review; summarize and disseminate research findings; and identify research gaps in the existing literature (Arksey and O'Malley, 2005; Levac *et al.*, 2010). As it provides a rigorous and transparent method for mapping areas of research, a scoping review can be used as a standalone project or as a preliminary step to a systematic review (Arksey and O'Malley, 2005).

Scoping reviews share a number of the same processes as systematic reviews as they both use rigorous and transparent methods to comprehensively identify and analyze all the relevant literature pertaining to a research question (DiCenso *et al.*, 2010). The key differences between the two review methods can be attributed to their differing purposes and aims. First, the purpose of a scoping review is to map the body of literature on a topic area (Arksey and O'Malley, 2005), whereas the purpose of a systematic review is to sum up the best available research on a specific question (Campbell Collaboration, 2013). Subsequently, a scoping review seeks to present an overview of a potentially large and diverse body of literature pertaining to a broad topic, whereas a systematic review attempts to collate empirical evidence from a relatively smaller number of studies pertaining to a focused research question (Arksey and O'Malley, 2005; Higgins and Green, 2011). Second, scoping reviews generally include a greater range of study designs and methodologies than systematic reviews addressing the effectiveness of interventions, which often focus on randomized controlled trials (Arksey and O'Malley, 2005). Third, scoping reviews aim to provide a descriptive overview of the reviewed material without critically appraising individual studies or synthesizing evidence from different studies (Arksey and O'Malley, 2005; Brien *et al.*, 2010). In contrast, systematic reviews aim to provide a synthesis of evidence from studies assessed for risk of bias (Higgins and Green, 2011).

Scoping reviews are a relatively new approach for which there is not yet a universal study definition or definitive procedure (Arksey and O'Malley, 2005; Anderson *et al.*, 2008; Davis *et al.*, 2009; Levac *et al.*, 2010; Daudt *et al.*, 2013). In 2005, Arksey and O'Malley published the first methodological framework for conducting scoping reviews with the aims of clarifying when and how one might be undertaken. They proposed an iterative six-stage process: (1) identifying the research question, (2) identifying relevant studies, (3) study selection, (4) charting the data, (5) collating, summarizing and reporting the results, and (6) an optional consultation exercise (Arksey and O'Malley, 2005). Arksey and O'Malley intended for their framework to stimulate discussion about the value of scoping reviews and provide a starting point toward a methodological framework. Since its publication, a few researchers have proposed enhancements to the Arksey and O'Malley framework based on their own experiences with it (Brien *et al.*, 2010; Levac *et al.*, 2010; Daudt *et al.*, 2013) or a review of a selection of scoping reviews (Anderson *et al.*, 2008; Davis *et al.*, 2009).

In recent years, scoping reviews have become an increasingly adopted approach and have been published across a broad range of disciplines and fields of study (Anderson *et al.*, 2008). To date, little has been published of the extent, nature, and use of completed scoping reviews. One study that explored the nature of scoping reviews within the nursing literature found that the included reviews (*N* = 24) varied widely in terms of intent, procedure, and methodological rigor (Davis *et al.*, 2009). Another study that examined 24 scoping reviews commissioned by a health research program found that the nature and type of the reports were wide ranging and reported that the value of scoping reviews is ‘increasingly limited by a lack of definition and clarity of purpose’ (Anderson *et al.*, 2008). Given that these studies examined only a small number of scoping reviews from select fields, it is not known to what extent scoping reviews have been undertaken in other fields of research and whether these findings are representative of all scoping reviews as a whole. A review of scoping reviews across the literature can provide a better understanding of how the approach has been used and some of the limitations and challenges encountered by scoping review authors. This information would provide a basis for the development and adoption of a universal definition and methodological framework.

The purpose of this paper is to provide an overview of existing scoping reviews in the literature. The four specific objectives of this scoping review were to (1) conduct a systematic search of the published and gray literature for scoping review papers, (2) map out the characteristics and range of methodologies used in the identified scoping reviews, (3) examine reported challenges and limitations of the scoping review approach, and (4) propose recommendations for advancing the approach and enhancing the consistency with which they are undertaken and reported.

## 2. Methods

This scoping review began with the establishment of a research team consisting of individuals with expertise in epidemiology and research synthesis (Levac *et al.*, 2010). The team advised on the broad research question to be addressed and the overall study protocol, including identification of search terms and selection of databases to search.

The methodology for this scoping review was based on the framework outlined by Arksey and O'Malley (2005) and ensuing recommendations made by Levac *et al*. (2010). The review included the following five key phases: (1) identifying the research question, (2) identifying relevant studies, (3) study selection, (4) charting the data, and (5) collating, summarizing, and reporting the results. The optional ‘consultation exercise’ of the framework was not conducted. A detailed review protocol can be obtained from the primary author upon request.

### 2.1. Research question

This review was guided by the question, ‘What are the characteristics and range of methodologies used in scoping reviews in the literature?’ For the purposes of this study, a scoping review is defined as a type of research synthesis that aims to ‘map the literature on a particular topic or research area and provide an opportunity to identify key concepts; gaps in the research; and types and sources of evidence to inform practice, policymaking, and research’ (Daudt *et al.*, 2013).

### 2.2. Data sources and search strategy

The initial search was implemented on June 17, 2011, in four electronic databases: MEDLINE/PubMed (biomedical sciences, 1946–present), SciVerse Scopus (multidisciplinary; 1823–present), CINAHL/EBSCO (nursing and allied health; 1981–present) and Current Contents Connect/ISI Web of Knowledge (multidisciplinary current awareness; 1998–present). The databases were selected to be comprehensive and to cover a broad range of disciplines. No limits on date, language, subject or type were placed on the database search. The search query consisted of terms considered by the authors to describe the scoping review and its methodology: scoping review, scoping study, scoping project, literature mapping, scoping exercise, scoping report, evidence mapping, systematic mapping, and rapid review. The search query was tailored to the specific requirements of each database (see Additional file 1).

Applying the same search string that was used for the search in SciVerse Scopus (Elsevier), a web search was conducted in SciVerse Hub (Elsevier) to identify gray literature. The *a priori* decision was made to screen only the first 100 hits (as sorted by relevance by Scopus Hub) after considering the time required to screen each hit and because it was believed that further screening was unlikely to yield many more relevant articles (Stevinson and Lawlor, 2004). The following websites were also searched manually: the Health Services Delivery Research Programme of the National Institute for Health Research (http://www.netscc.ac.uk/hsdr/), the National Co-ordinating Centre for NHS Service Delivery and Organisation (http://php.york.ac.uk/inst/spru/pubs/main.php), NHS Evidence by the National Institute for Health and Clinical Excellence (http://evidence.nhs.uk/), the University of York Social Policy Research Unit (http://php.york.ac.uk/inst/spru/pubs/main.php), the United Kingdom's Department of Health (http://www.dh.gov.uk/en/index.htm), and Google (http://www.google.com).

The reference lists of 10 randomly selected relevant articles (Hazel, 2005; Vissandjee *et al.*, 2007; Gagliardi *et al.*, 2009; Meredith *et al.*, 2009; Bassi *et al.*, 2010; Ravenek *et al.*, 2010; Sawka *et al.*, 2010; Churchill *et al.*, 2011; Kushki *et al.*, 2011; Spilsbury *et al.*, 2011) and eight review articles on scoping reviews (Arksey and O'Malley, 2005; Anderson *et al.*, 2008; Davis *et al.*, 2009; Grant and Booth, 2009; Hetrick *et al.*, 2010; Levac *et al.*, 2010; Rumrill *et al.*, 2010; Armstrong *et al.*, 2011) were manually searched to identify any further scoping reviews not yet captured. A ‘snowball’ technique was also adopted in which citations within articles were searched if they appeared relevant to the review (Hepplestone *et al.*, 2011; Jaskiewicz and Tulenko, 2012).

A follow-up search of the four bibliographic databases and gray literature sources was conducted on October 1, 2012 to identify any additional scoping reviews published after the initial search [see Additional file 1]. A search of Google with no date restrictions was also conducted at this time; only the first 100 hits (as sorted by relevance by Google) were screened.

### 2.3. Citation management

All citations were imported into the web-based bibliographic manager RefWorks 2.0 (RefWorks-COS, Bethesda, MD), and duplicate citations were removed manually with further duplicates removed when found later in the process. Citations were then imported into the web-based systematic review software DistillerSR (Evidence Partners Incorporated, Ottawa, ON) for subsequent title and abstract relevance screening and data characterization of full articles.

### 2.4. Eligibility criteria

A two-stage screening process was used to assess the relevance of studies identified in the search. Studies were eligible for inclusion if they broadly described the use of a scoping review methodology to identify and characterize the existing literature or evidence base on a broad topic. Because of limited resources for translation, articles published in languages other than English, French, or Spanish were excluded. Papers that described the scoping review process without conducting one and reviews of scoping reviews were excluded from the analysis, but their reference list was reviewed to identify additional scoping reviews. When the same data were reported in more than one publication (e.g., in a journal article and electronic report), only the article reporting the most complete data set was used.

### 2.5. Title and abstract relevance screening

For the first level of screening, only the title and abstract of citations were reviewed to preclude waste of resources in procuring articles that did not meet the minimum inclusion criteria. A title and abstract relevance screening form was developed by the authors and reviewed by the research team (see Additional file 2). The form was pretested by three reviewers (M. P., J. G., I. Y.) using 20 citations to evaluate reviewer agreement. The overall kappa of the pretest was 0.948, where a kappa of greater than 0.8 is considered to represent a high level of agreement (Dohoo *et al.*, 2012). As there were no significant disagreements among reviewers and the reviewers had no revisions to recommend, no changes were made to the form. The title and abstract of each citation were independently screened by two reviewers. Reviewers were not masked to author or journal name. Titles for which an abstract was not available were included for subsequent review of the full article in the data characterization phase. Reviewers met throughout the screening process to resolve conflicts and discuss any uncertainties related to study selection (Levac *et al.*, 2010). The overall kappa was 0.90.

### 2.6. Data characterization

All citations deemed relevant after title and abstract screening were procured for subsequent review of the full-text article. For articles that could not be obtained through institutional holdings available to the authors, attempts were made to contact the source author or journal for assistance in procuring the article. A form was developed by the authors to confirm relevance and to extract study characteristics such as publication year, publication type, study sector, terminology, use of a published framework, quality assessment of individual studies, types of data sources included, number of reviewers, and reported challenges and limitations (see Additional file 3). This form was reviewed by the research team and pretested by all reviewers (M. P., A. R., J. G., I. Y., K. G.) before implementation, resulting in minor modifications to the form. The characteristics of each full-text article were extracted by two independent reviewers (M. P. and J. G./K. G.). Studies excluded at this phase if they were found to not meet the eligibility criteria. Upon independently reviewing a batch of 20 to 30 articles, the reviewers met to resolve any conflicts and to help ensure consistency between reviewers and with the research question and purpose (Levac *et al.*, 2010).

### 2.7. Data summary and synthesis

The data were compiled in a single spreadsheet and imported into Microsoft Excel 2010 (Microsoft Corporation, Redmond, WA) for validation and coding. Fields allowing string values were examined for implausible values. The data were then exported into STATA version 12 (StataCorp, College Station, TX) for analyses. Descriptive statistics were calculated to summarize the data. Frequencies and percentages were utilized to describe nominal data.

## 3. Results

### 3.1. Search and selection of scoping reviews

**Figure 1:**
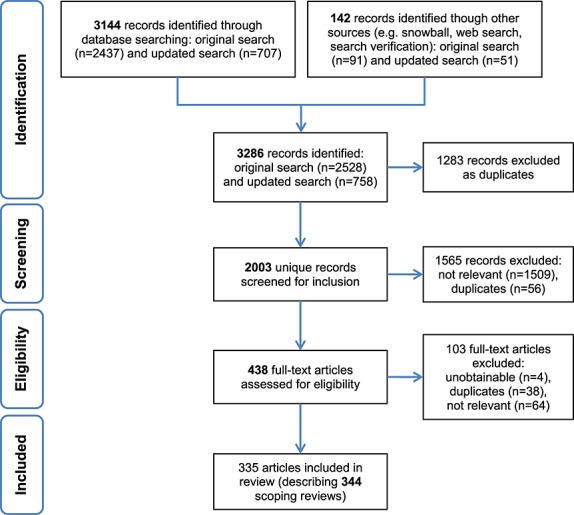
PRISMA flowchart of study selection process.

The original search conducted in June 2011 yielded 2528 potentially relevant citations. After deduplication and relevance screening, 238 citations met the eligibility criteria based on title and abstract and the corresponding full-text articles were procured for review. Four articles could not be procured and were thus not included in the review (Levy and Sanghvi, 1986; Bhavaraju, 1987; Centre for Reviews and Dissemination, 2004; Connell *et al.*, 2006). After data characterization of the full-text articles, 182 scoping reviews remained and were included in the analysis. The updated search in October 2012 produced 758 potentially relevant citations and resulted in another 162 scoping reviews being included. In total, 344 scoping reviews were included in the study. The flow of articles through identification to final inclusion is represented in Figure 1.

Many citations were excluded upon screening at the title and abstract level as several terms used in the search algorithm also corresponded to other study designs. For example, the term ‘scoping study’ was also used to describe studies that assessed the chemical composition of samples (e.g., Behrens *et al.*, 1998; Banks and Banks, 2001; Forrest *et al.*, 2011) and preliminary mining studies (Butcher, 2002; Bhargava *et al.*, 2008). ‘Scoping exercise’ also described studies that scoped an issue using questionnaires, focus groups, and/or interviews (e.g., Malloch and Burgess, 2007; Willis *et al.*, 2011; Norwood and Skinner, 2012). ‘Rapid review’ was also used to describe the partial rescreening of negative cervical smears as a method of internal quality assurance (e.g., Faraker and Boxer, 1996; Frist, 1997; Shield and Cox, 1998). ‘Systematic mapping’ was also used in studies pertaining to topographic mapping (e.g., Noda and Fujikado, 1987; Gunnell, 1997; Liu *et al.*, 2011) and mapping of biomolecular structures (e.g., Camargo *et al.*, 1976; Descarries *et al.*, 1982; Betz *et al.*, 2006).

### 3.2. General characteristics of included scoping reviews

The general characteristics of scoping reviews included in this study are reported in Table 1. All included reviews were published between 1999 and October 2012, with 68.9% (237/344) published after 2009. Most reviews did not report the length of time taken to conduct the review; for the 12.8% (44/344) that did, the mean length was approximately 5.2 months with a range of 2 weeks to 20 months. Journal articles (64.8%; 223/344) and government or research station reports (27.6%; 95/344) comprised the majority of documents included in the review. The number of journal articles was slightly underrepresented as 10 were excluded as duplicates because the same scoping review was also reported in greater detail in a report. The included reports ranged greatly in length, from four pages (Healthcare Improvement Scotland, 2012) to over 300 pages (Wallace *et al.*, 2006).

**Table 1:** General characteristics of included scoping reviews (*n* = 344)

Characteristic	Number (*n* = 344)	Percentage (%)
Publication year		
<2000	1	0.3
2000–2004	19	5.5
2005–2009	87	25.3
2010–October 2012	237	68.9
Publication type		
Journal article	223	64.8
Conference proceeding	25	7.3
Thesis dissertation	1	0.3
Government or research station report	95	27.6
Sector		
Health	202	58.7
Health and Social sciences	53	15.4
Social sciences	14	4.1
Business	1	0.3
Agriculture and agri-food	4	1.2
Education	15	4.4
Software engineering	41	11.9
Other	14	4.1
Scoping terminology		
Scoping review	212	61.6
Scoping study	42	12.2
Systematic mapping	42	12.2
Evidence mapping	9	2.6
Literature mapping	4	1.2
Rapid review	5	1.5
Scoping exercise	15	4.4
Other	15	4.4
Scoping definition		
Reported in article	217	63.1
Not provided, cited another source	22	6.4
Study length (mean; range)	5.15 months	2 weeks to 20 months

The included scoping reviews varied widely in terms of the terminology used to describe the methodology. ‘Scoping review’ was the term most often used, reported in 61.6% (212/344) of included studies. An explicit definition or description of what study authors meant by ‘scoping review’ was reported in 63.1% (217/344) of articles. Most definitions centered around scoping reviews as a type of literature that identifies and characterizes, or maps, the available research on a broad topic. However, there was some divergence in how study authors characterized the rigor of the scoping review methodology. The terms ‘systematic’, ‘rigorous’, ‘replicable’, and ‘transparent’ were frequently used to describe the methodology, and several authors described scoping reviews to be comparable in rigor to systematic reviews (Gagliardi *et al.*, 2009; Liu *et al.*, 2010; Ravenek *et al.*, 2010; Feehan *et al.*, 2011; Heller *et al.*, 2011). In contrast, some studies described the methodology as less rigorous or systematic than a systematic review (Cameron *et al.*, 2008; Levac *et al.*, 2009; Campbell *et al.*, 2011). Brien *et al.* (2010) commented that scoping reviews were ‘often misinterpreted to be a less rigorous systematic review, when in actual fact they are a different entity’.

Some reviews were conducted as stand-alone projects while others were undertaken as parts of larger research projects. Study authors reported that a main purpose or objective for the majority of articles (97.4%; 335/344) was to identify, characterize, and summarize research evidence on a topic, including identification of research gaps. Only 6.4% (22/344) of included articles conducted the scoping review methodology to identify questions for a systematic review. As response options were not mutually exclusive, some reviews reported multiple purposes and/or objectives. A commissioning source was reported in 31.4% (108/344) of reviews; some reported that they were specifically commissioned to advise a funding body as to what further research should be undertaken in an area (e.g., Arksey *et al.*, 2002; Carr-Hill *et al.*, 2003; Fotaki *et al.*, 2005; Baxter *et al.*, 2008; Williams *et al.*, 2008; Trivedi *et al.*, 2009; Crilly *et al.*, 2010; Brearley *et al.*, 2011).

The majority of the included scoping reviews addressed a health topic, making up 74.1% (255/344) of reviews. The use of scoping reviews in software engineering—or ‘systematic mapping’ as termed in the sector—has increased in recent years with 92.7% (38/41) published after 2010. The topics examined in the included scoping reviews ranged greatly, spanning from data on multiplayer online role-playing games (Meredith *et al.*, 2009), to factors that influence antibiotic prophylaxis administration (Gagliardi *et al.*, 2009). The topics investigated were generally broad in nature, such as ‘what is known about the diagnosis, treatment and management of obesity in older adults’ (Decaria *et al.*, 2012). Some reviews that were conducted under short time frames (e.g., 1 month) addressed more specific questions such as ‘what is the published evidence of an association between hospital volume and operative mortality for surgical repair (open and endovascular) of unruptured and ruptured abdominal aortic aneurysms?’ (Healthcare Improvement Scotland, 2011).

### 3.3. Methodological characteristics of included scoping reviews

The methodological characteristics of included scoping reviews are reported in Table 2. Approximately half of the reviews (50.6%; 174/344) reported using one or more methodological frameworks for carrying out the scoping review. Framework use varied greatly between reviews from different sectors, such as in 85.4% (35/41) of reviews from the software engineering sector and in 44.0% (89/202) of health sector reviews. Overall, the Arksey and O'Malley (2005) framework was the most frequently used, reported in 62.6% (109/174) of studies that reported using a framework. Among reviews from the software engineering sector that reported using a framework, frameworks by Kitchenham and Charters (2007) (40.0%; 14/35) and Petersen *et al*. (2008) (51.4%; 18/35) were most commonly employed. The use of a framework increased over time, from 31.6% (6/19) of reviews published from 2000 to 2004, to 42.5% (37/87) of reviews from 2005 to 2009, and to 55.3% (131/237) of reviews published from 2010 onward.

**Table 2:** Methodological characteristics of included reviews (*n* = 344)

Methodological characteristic	Number (*n* = 344)	Percentage (%)
General methodology		
Used a published framework	174	50.6
Consulted stakeholders	164	47.7
Conducted quality assessment	77	22.4
Search strategy		
Searched electronic database(s)	332	96.5
Searched reference list of relevant articles	170	49.4
Manual searching of select journals	94	27.3
Search in Internet search engines or specific websites	149	43.3
Consulted experts	99	28.8
Performed an updated search	24	7.0
Study selection		
Used defined inclusion/exclusion criteria	274	79.7
Screening of titles and abstracts by ≥2 reviewers	88	25.6
Screening of full-text articles by ≥2 reviewers	68	19.8
No limits on study design	252	73.3
Limited to controlled trials only	10	2.9
No limits on publication type	201	58.4
Limited to peer-reviewed articles	42	12.2
Limited to journal articles (peer and non-peer-reviewed)	83	24.1
Data charting		
Data extraction by one reviewer	31	9.0
Data extraction by one reviewer, responses verified by another reviewer	41	11.9
Data extraction by ≥2 reviewers	62	18.0
Use of a standardized form	243	70.6
Data Analysis		
Number of articles included (min, max)	0	5258
Descriptive narrative summary	344	100
Formal qualitative analysis	21	5.8
Meta-analysis	0	0.0

Following the search, 79.7% (174/344) of reviews used defined inclusion and exclusion criteria to screen out studies that were not relevant to the review question(s). Among these, only six reviews explicitly reported that criteria were redefined or amended on a *post hoc* basis during the review process (While *et al.*, 2005; Marsella, 2009; Crooks *et al.*, 2010; Johnston *et al.*, 2010; Snyder *et al.*, 2011; Victoor *et al.*, 2012). The selection criteria in a few reviews were unclear due to ambiguous wording such as ‘real paper’ (Saraiva *et al.*, 2012), ‘scientific papers’ (Victoor *et al.*, 2012), and ‘culling low-interest articles’ (Catts *et al.*, 2010). Compared with the study selection process, fewer details were generally reported about the data characterization (or charting) of individual studies. Nearly a quarter of reviews (23.8%; 82/344) did not report any detail as to how the included studies were characterized, and it was unclear in 33.4% (115/344) as to how many reviewers were involved.

The majority of included reviews (77.7%, 267/344) did not assess the methodological quality of individual studies. A number of these studies reported that quality assessment was not conducted as it is not a priority in scoping reviews or part of the scoping review methodology. Two studies reported the use of publication in a peer-reviewed publication as a proxy for good quality (Baxter *et al.*, 2008; Pita *et al.*, 2011) and another reported using studies included in existing reviews or meta-analyses to ‘overcome’ the lack of quality assessment (MacDougall, 2011). Of the 22.4% (77/344) of articles that reported a critical appraisal step, the rigor with which it was conducted ranged from the reviewer's subjective assessment using a scale of high, medium, or low (Roland *et al.*, 2006), to the use of published tools such as the Jadad scale (Jadad *et al.*, 1996) for randomized control trials (Deshpande *et al.*, 2009; Borkhoff *et al.*, 2011).

The level of detail reported about the search strategy varied considerably across the reviews. Table 3 displays information about the search strategy reported in the included reviews by time. Overall, the detail of reporting for the search increased numerically over time. For example, 78.06% of reviews published after 2009 reported complete strings or a complete list of search terms, compared with 57.89% of reviews published between 2000 and 2004 and 67.82% of reviews published between 2005 and 2009.

**Table 3:** Search strategy details reported in included reviews, by year

	<2000 (*n* = 1)	2000–2004 (*n* = 19)	2005–2009 (*n* = 87)	2010–Oct 2012 (*n* = 237)	Total (*n* = 344)
Search terms	0%	57.89%	67.82%	78.06%	74.13%
Search period	100%	84.21%	72.41%	77.64%	76.74%
Search limits	0%	63.16%	72.41%	79.32%	76.45%
Search date	0%	47.37%	48.28%	57.38%	54.36%
Updated search	0%	0%	2.30%	9.28%	6.98%
Data sources	100%	84.21%	90.80%	91.56%	90.99%
In appendix	0%	31.58%	37.93%	28.69%	31.10%

**Figure 2:**
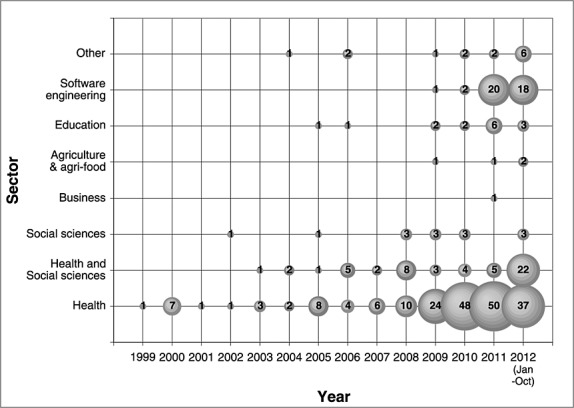
Bubble plot of scoping reviews published by year and sector. The size of a bubble is proportional to the number of scoping reviews published in the year and sector corresponding to the bubble coordinates.

Table 4 summarizes how some of the results of the included reviews were reported and ‘charted’. A flow diagram was used to display the flow of articles from the initial search to final selection in 35.8% of reviews (123/344). Characteristics of included studies were often displayed in tables (82.9%; 285/344), ranging from basic tables that described the key characteristics of each included study, to cross-tabulation heat maps that used color-coding to highlight cell values. Study characteristics were also mapped graphically in 28.8% (99/344) of reviews, often in the form of histograms, scatterplots, or pie charts. Reviews from the software engineering sector frequently used bubble charts to map the data (Figure 2 is an example of a bubble chart). In summarizing the reviewed literature, 77.6% (267/344) of reviews noted gaps where little or no research had been conducted, and 77.9% (268/344) recommended topics or questions for future research.

**Table 4:** Reporting of results the included scoping reviews

	Number (*n* = 344)	Percentage (%)
Depiction of flow of articles from search to final selection		
Narrative text	247	71.8
Flow diagram (e.g., PRISMA)	123	35.8
Table	20	5.8
Charting of included studies		
Tabular format	285	82.9
Graphical format	99	28.8
Implications of findings		
Identified gaps in the research	267	77.6
Recommended topics or questions for future research	268	77.9
Recommended a systematic review be conducted	34	9.9
Inform design or scope of future research	11	3.2
Policy implications or recommendations for policy or practice	63	18.3

Stakeholder consultation is an optional sixth-step in the Arksey and O'Malley (2005) framework and was reported in 39.8% (137/344) of reviews. This optional step was reported in 34.9% (38/109) of reviews that used the Arksey and O'Malley framework, compared with 42.13% (99/235) of reviews that did not. Stakeholders were most often consulted at the search phase to assist with keyword selection for the search strategy or help identify potential studies to include in the review (74.5%; 102/137). Stakeholders were less frequently involved in the interpretation of research findings (30.7%; 42/137) and in the provision of comments at the report writing stage (24.1%; 33/137). Ongoing interaction with stakeholders throughout the review process was reported in 25.9% (89/344) of all reviews. Comparing between sectors, the proportion of reviews that reported consulting with stakeholders was highest in the social sciences sector (71.4%; 10/14) and lowest in the software engineering sector (2.4%; 1/41).

### 3.4. Reported challenges and limitations

Limitations in the study approach were reported in 71.2% (245/344) of reviews. The most frequent limitation reported in the reviews was the possibility that the review may have missed some relevant studies (32.0%; 110/344). This limitation was frequently attributed to database selection (i.e., searching other databases may have identified additional relevant studies), exclusion of the gray literature from the search, time constraints, or the exclusion of studies published in a language other than English. In comparison with systematic reviews, one review noted that it was ‘unrealistic to retrieve and screen all the relevant literature’ in a scoping review due to its broader focus (Gentles *et al.*, 2010), and a few noted that all relevant studies may not have been identified as scoping reviews are not intended to be as exhaustive or comprehensive (Cameron *et al.*, 2008; Levac *et al.*, 2009; Boydell *et al.*, 2012).

The balance between breadth and depth of analysis was a challenge reported in some reviews. Brien *et al.* (2010) and Cronin de Chavez *et al*. (2005) reported that it was not feasible to conduct a comprehensive synthesis of the literature given the large volume of articles identified in their reviews. Depth of analysis was also reported to be limited by the time available to conduct the review (Freeman *et al.*, 2000; Gulliford *et al.*, 2001; Templeton *et al.*, 2006; Cahill *et al.*, 2008; Bostock *et al.*, 2009; Brodie *et al.*, 2009).

The lack of critical appraisal of included studies was reported as a study limitation in 16.0% (55/344) of reviews. One review commented that this was the primary limitation of scoping reviews (Feehan *et al.*, 2011), and others noted that without this step, scoping reviews cannot identify gaps in the literature related to low quality of research (Hand and Letts, 2009; Brien *et al.*, 2010). Additionally, two reviews reported that their results could not be used to make recommendations for policy or practice because they did not assess the quality of included studies (Bostrom *et al.*, 2011; Churchill *et al.*, 2011). Conversely, Njelesani *et al*. (2011) noted that ‘by not addressing the issues of quality appraisal, this study dealt with a greater range of study designs and methodologies than would have been included in a systematic review’, and McColl *et al.* (2009) commented that ‘the emphasis of a scoping study is on comprehensive coverage, rather than on a particular standard of evidence’.

## 4. Discussion

In this paper, we provided an overview of scoping reviews identified in the gray and published literature. Our search for scoping reviews in the published and gray literature aimed to be comprehensive while also balancing practicality and available resources. It was not within the remit of this scoping review to assess the methodological quality of individual scoping reviews included in the analysis. Based on the characteristics, range of methodologies and reported challenges in the included scoping reviews, we have proposed some recommendations for advancing the scoping review approach and enhancing the consistency with which they are undertaken and reported.

### 4.1. Overview of included scoping reviews

Our results corroborate that scoping reviews are a relatively new approach that has gained momentum as a distinct research activity in recent years. The identified reviews varied in terms of terminology, purpose, methodological rigor, and level of detail of reporting; therefore, there appears to be a lack of clarity or agreement around the appropriate methodology for scoping reviews. In a scoping review that reviewed 24 scoping reviews from the nursing literature, Davis *et al.* (2009) also reported that the included scoping reviews varied widely in terms of intent, procedural, and methodological rigor. Given that scoping reviews are a relatively new methodology for which there is not yet a universal study definition, definitive procedure or reporting guidelines, the variability with which scoping reviews have been conducted and reported to date is not surprising. However, efforts have been made by scoping review authors such as Arksey and O'Malley (2005); Anderson *et al.* (2008); Davis *et al.* (2009); Brien *et al.* (2010); Levac *et al.* (2010) and Daudt *et al.* (2013) to guide other researchers in undertaking and reporting scoping reviews, as well as clarifying, enhancing, and standardizing the methodology. Their efforts seem to be having some impact given the increase in the number of scoping reviews disseminated in the published and gray literature, the growth in the use of a methodological framework, and the greater amount of detail and consistency with which scoping review processes have been reported.

### 4.2. Recommendations

Levac *et al.* (2010) remarked that discrepancies in nomenclature between ‘scoping reviews’, ‘scoping studies’, ‘scoping literature reviews’, ‘scoping exercises’, and so on lead to confusion, and consequently used the term ‘scoping study’ for consistency with the Arksey and O'Malley framework. We agree that there is a need for consistency in terminology; however, we argue that the term ‘scoping review’ should be adopted in favor of ‘scoping study’ or the other terms that have been used to describe the method. Our review has found that ‘scoping review’ is the most commonly used term in the literature to denote the methodology and that a number of the other terms (i.e., scoping study, scoping exercise, and systematic mapping) have been used to describe a variety of primary research study designs. Furthermore, we find that the word ‘review’ more explicitly indicates that the term is referring to a type of literature review, compared with ‘study’ or ‘exercise’.

As scoping reviews share many of the same processes with the more commonly known systematic review, many of the included reviews compared and contrasted the two methods. We concur with Brien *et al.* (2010) that scoping reviews are often misinterpreted as a less rigorous version of a systematic review, when in fact they are a ‘different entity’ with a different set of purposes and objectives. We contend that researchers adopting a systematic review approach but with concessions in rigor to shorten the timescale, refer to the process as a ‘rapid review’. Scoping reviews are one method among many available to reviewing the literature (Arksey and O'Malley, 2005), and researchers need to consider their research question or study purpose when deciding which review approach is most appropriate. Additionally, given that some of the included reviews took over 1 year to complete, we agree that it would be wrong to necessarily assume that scoping reviews represent a quick alternative to a systematic review (Arksey and O'Malley, 2005).

There is an ongoing deliberation in the literature regarding the need for quality assessment of included studies in the scoping review process. While Arksey and O'Malley stated that ‘quality assessment does not form part of the scoping (review) remit’, they also acknowledged this to be a limitation of the method. This may explain why quality assessment was infrequently performed in the included reviews and why it was reported as a study limitation among a number of these reviews. In their follow-up recommendations to the Arksey and O'Malley framework, Levac *et al.* (2010) did not take a position on the matter but recommended that the debate on the need for quality assessment continue. However, a recent paper by Daudt *et al.* (2013) asserts that it is a necessary component of scoping reviews and should be performed using validated tools. We argue that scoping reviews should include all relevant literature regardless of methodological quality, given that their intent is to present an overview of the existing literature in a field of interest without synthesizing evidence from different studies (Arksey and O'Malley, 2005). In doing so, scoping reviews can provide a more complete overview of all the research activity related to a topic. However, we also recognize that some form of quality assessment of all included studies would enable the identification of gaps in the evidence base—and not just where research is lacking—and a better determination of the feasibility of a systematic review. The debate on the need for quality assessment should consider the challenges in assessing quality among the wide range of study designs and large volume of literature that can be included in scoping reviews (Levac *et al.*, 2010).

The lack of consistency among the included reviews was not surprising given the lack of a universal definition or purpose for scoping reviews (Anderson *et al.*, 2008; Davis *et al.*, 2009; Levac *et al.*, 2010; Daudt *et al.*, 2013). The most commonly cited definition scoping reviews may be the one set forth by Mays *et al*. (2001) and used by Arksey and O'Malley: ‘scoping studies aim to map *rapidly* the key concepts underpinning a research area and the main sources and types of evidence available and can be undertaken as standalone projects in their own right, especially where an area is complex or has not been reviewed extensively before’. However, we believe that a recently proposed definition by Daudt *et al*. (2013) is more straightforward and fitting of the method: ‘scoping studies aim to map the literature on a particular topic or research area and provide an opportunity to identify key concepts; gaps in the research; and types and sources of evidence to inform practice, policymaking, and research’. While we would replace the term ‘scoping studies’ with ‘scoping reviews’, we endorse the Daudt *et al*. definition because it clearly articulates that scoping reviews are a type of literature review and removes the emphasis away from being ‘rapid’ process.

It has been suggested that the optimal scoping review is ‘one that demonstrates procedural and methodological rigor in its application’ (Davis *et al.*, 2009). We found that some scoping reviews were not reported in sufficient detail to be able to demonstrate ‘rigor in its application’. When there is a lack of clarity or transparency relating to methodology, it is difficult to distinguish poor reporting from poor design. We agree that it is crucial for scoping review authors to clearly report the processes and procedures undertaken—as well as any limitations of the approach—to ensure that readers have sufficient information to determine the value of findings and recommendations (Arksey and O'Malley, 2005; Davis *et al.*, 2009). The development of reporting guidelines for scoping reviews would help to ensure the quality and transparency of those undertaken in the future (Brien *et al.*, 2010). Given that reporting guidelines do not currently exist for scoping reviews (Brien *et al.*, 2010), researchers conducting scoping reviews may want to consider using the Preferred Reporting Items for Systematic Reviews and Meta-Analyses (http://prisma-statement.org/) as a guide, where applicable.

### 4.3. Strengths and limitations of this scoping review

This scoping review used rigorous and transparent methods throughout the entire process. It was guided by a protocol reviewed by a research team with expertise in knowledge synthesis and scoping reviews. To ensure a broad search of the literature, the search strategy included four electronic bibliographic databases, the reference list of eighteen different articles, two internet search engines, the websites of relevant organizations, and the snowball technique. The relevance screening and data characterization forms were pretested by all reviewers and revised as needed prior to implementation. Each citation and article was reviewed by two independent reviewers who met in regular intervals to resolve conflicts. Our use of a bibliographic manager (RefWorks) in combination with systematic review software (DistillerSR) ensured that all citations and articles were properly accounted for during the process. Furthermore, an updated search was performed in October 2012 to enhance the timeliness of this review.

This review may not have identified all scoping reviews in the published and gray literature despite attempts to be as comprehensive as possible. Our search algorithm included nine different terms previously used to describe the scoping process; however, other terms may also exist. Although our search included two multidisciplinary databases (i.e., Scopus, Current Contents) and Google, the overall search strategy may have been biased toward health and sciences. Searching other bibliographic databases may have yielded additional published scoping reviews. While our review included any article published in English, French or Spanish, our search was conducted using only English terms. We may have missed some scoping reviews in the gray literature as only the first 100 hits from each Web search were screened for inclusion. Furthermore, we did not contact any researchers or experts for additional scoping reviews we may have missed.

Other reviewers may have included a slightly different set of reviews than those included in this present review. We adopted Arksey and O'Malley's definition for scoping reviews at the outset of the study and found that their simple definition was generally useful in guiding study selection. However, we encountered some challenges during study selection with reviews that also reported processes or definitions more typically associated with narrative, rapid or systematic reviews. We found that some reviews blurred the line between narrative and scoping reviews, between scoping and rapid reviews, and between scoping and systematic reviews. Our challenges echoed the questions: ‘where does one end and the other start?’ (Arksey and O'Malley, 2005) and ‘who decides whether a particular piece of work is a scoping (review) or not?’ (Anderson *et al.*, 2008). For this review, the pair of reviewers used their judgment to determine whether each review as a whole sufficiently met our study definition of a scoping review. On another note, characterization and interpretation of the included reviews were also subject to reviewer bias.

## 5. Conclusions

This scoping review of scoping reviews characterized and described the nature of scoping reviews in the published and gray literature. Scoping reviews are a relatively new approach to reviewing the literature, which has increased in popularity in recent years. As the purpose, methodological process, terminology, and reporting of scoping reviews have been highly variable, there is a need for their methodological standardization to maximize the utility and relevance of their findings. We agree that the establishment of a common definition and purpose for scoping reviews is an important step toward enhancing the consistency with which they are conducted (Levac *et al.*, 2010); this would provide a common platform from which debates regarding the methodology can ensue, and the basis for future methodological frameworks and reporting guidelines. We hope that the results of our study can contribute to the ongoing collective work of a number of researchers to further clarifying and enhancing the scoping review methodology.

## Supporting Information

Supporting info item

Supporting info item

Supporting info item

Supporting info item
