# Supplementary material for: A scoping review of scoping reviews: advancing the approach and enhancing the consistency
Source: Res Synth Methods. 2014 Jul 24;5(4):371–85. doi: 10.1002/jrsm.1123 (PMC4491356; doi:10.1002/jrsm.1123)
Supplement: Supplementary file 1 — Supporting info item [file jrsm0005-0371-sd1.pdf]

Additional file 1: Search details

Original search

A. Electronic databases

**Database/platform:**	**SciVerse Scopus (Elsevier)**
**Date coverage:**	1823 to present (greater than half of which are from 1996 to present)
**Library:**	Public Health Agency of Canada & Health Canada Libraries
**Date of search:**	Friday, June 17, 2011
**Limits:**	In: "Article Title, Abstract, Keywords"<br>Published: "All years" to "Present"<br>Document type: "All"<br>Subject Areas: All checked (default)
**Search query:**	"scoping study" OR "scoping review" OR "scoping project" OR "literature scoping" OR "literature mapping" OR "scoping exercise" OR "scoping report" OR "evidence map" OR "evidence mapping" OR "systematic map" OR "systematic mapping" OR "rapid review"
**Number of hits:**	1100

**Database/platform:**	**MEDLINE (PubMed)**
**Time coverage:**	Generally 1946 to present
**Library:**	Free access
**Date of search:**	Friday, June 17, 2011
**Limits:**	In: "All fields"<br>Date range: All years
**Search query:**	scoping stud* OR scoping review* OR scoping project* OR literature map* OR scoping exercise* OR scoping report* OR evidence map* OR systematic map* OR rapid review*
**Number of hits:**	454

**Database/platform:**	**CINAHL® (EBSCO)**
**Time coverage:**	Full text coverage from 1981 to present
**Library:**	University of Guelph
**Date of search:**	Friday, June 17, 2011
**Limits:**	In: "Select a field (optional)"<br>Search mode: "Boolean/Phrase"<br>Apply related words: [checked]<br>Limit your results: [None, all unchecked or left blank (Default)]
**Search query:**	"scoping stud*" OR "scoping review*" OR "scoping project*" OR "literature map*" OR "scoping exercise*" OR "scoping report*" OR "evidence map*" OR "systematic map*" OR "rapid review*"
**Number of hits:**	133

**Database/platform:**	**Current Contents Connect® (ISI Web of Knowledge)**
**Time coverage:**	1998 to present
**Library:**	University of Guelph
**Date of search:**	Friday, June 17, 2011
**Limits:**	In: "Topic"<br>Timespan: "All years"<br>Databases: ABES, SBS, CM, LS, PCES, ECT, AH
**Search query:**	"scoping study" OR "scoping studies" OR "scoping review*" OR "literature scoping" OR "literature mapping" OR "literature map" OR "scoping project*" OR "scoping exercise*" OR "evidence mapping" OR "systematic map" OR

	"systematic mapping" OR "rapid review"
**Number of hits:**	750

B. Web search and websites

**Database/platform:**	**SciVerse Scopus Web (Elsevier)**
**Library:**	Public Health Agency of Canada & Health Canada Libraries
**Date of search:**	Wednesday, July 6, 2011
**Limits:**	In: "Article Title, Abstract, Keywords"<br>Published: "All years" to "Present"<br>Document type: "All"<br>Subject Areas: All checked (default)
**Search query:**	"scoping study" OR "scoping review" OR "scoping project" OR "literature scoping" OR "literature mapping" OR "scoping exercise" OR "scoping report" OR "evidence map" OR "evidence mapping" OR "systematic map" OR "systematic mapping" OR "rapid review"
**Number of hits:**	92,924
**Notes:**	Reviewed first 100 hits

**Website:**	**National Institute for Health Research (NIHR)<br>Service Delivery and Organisation Programme (SDO)<br>Health Services and Delivery Research Programme**
**URL:**	http://www.sdo.nihr.ac.uk/newspublicationsandevents.html
**Date of search:**	Friday, July 8, 2011

**Website:**	**The University of York Social Policy Research Unit**
**URL:**	http://php.york.ac.uk/inst/spru/pubs/main.php
**Date of search:**	Friday, July 8, 2011

C. Reference lists

Articles on scoping reviews:

1. Anderson, S., Allen, P., Peckham, S., & Goodwin, N. (2008). Asking the right questions: Scoping studies in the commissioning of research on the organisation and delivery of health services. Health Research Policy and Systems / BioMed Central, 6, 7. doi:10.1186/1478-4505-6-7

2. Armstrong, R., Hall, B. J., Doyle, J., & Waters, E. (2011). 'Scoping the scope' of a Cochrane review. Journal of Public Health, 33(1), 147-150.

3. Arksey, H., & O'Malley, L. (2005). Scoping studies: Towards a methodological framework. International Journal of Social Research Methodology: Theory and Practice, 8(1), 19-32. doi:10.1080/1364557032000119616

4. Davis, K., Drey, N., & Gould, D. (2009). What are scoping studies? A review of the nursing literature. International Journal of Nursing Studies, 46(10), 1386-1400. doi:10.1016/j.ijnurstu.2009.02.010

5. Grant, M. J., & Booth, A. (2009). A typology of reviews: An analysis of 14 review types and associated methodologies. Health Information and Libraries Journal, 26(2), 91-108.

6. Hetrick, S. E., Parker, A. G., Callahan, P., & Purcell, R. (2010). Evidence mapping: Illustrating an emerging methodology to improve evidence-based practice in youth mental health. Journal of Evaluation in Clinical Practice, 16(6), 1025-1030.

7. Levac, D., Colquhoun, H., & O'Brien, K. K. (2010). Scoping studies: Advancing the methodology. Implementation Science, 5(1). doi:10.1186/1748-5908-5-69

8. Rumrill, P. D., Fitzgerald, S. M., & Merchant, W. R. (2010). Using scoping literature reviews as a means of understanding and interpreting existing literature. Work, 35(3), 399-404. doi:10.3233/WOR-2010-0998

Randomly-selected scoping review articles:

1. Bassi, J., Lau, F., & Bardal, S. (2010). Use of information technology in medication reconciliation: A scoping review. *Annals of Pharmacotherapy, 44*(5), 885-897. doi:10.1345/aph.1M699

2. Churchill, P., Otal, D., Pemberton, J., Ali, A., Flageole, H., & Walton, J. M. (2011). Sclerotherapy for lymphatic malformations in children: A scoping review. *Journal of Pediatric Surgery, 46*(5), 912-922. doi:10.1016/j.jpedsurg.2011.02.027

3. Gagliardi, A. R., Fenech, D., Eskicioglu, C., Nathens, A. B., & McLeod, R. (2009). Factors influencing antibiotic prophylaxis for surgical site infection prevention in general surgery: A review of the literature. *Canadian Journal of Surgery, 52*(6), 481-489. Retrieved from SCOPUS database.

4. Hazel, N. (2005). Holidays for children and families in need: An exploration of the research and policy context for social tourism in the UK. *Children and Society, 19*(3), 225-236. doi:10.1002/chi.838

5. Kushki, A., Chau, T., & Anagnostou, E. (2011). Handwriting difficulties in children with autism spectrum disorders: A scoping review. *Journal of Autism and Developmental Disorders*, , 1-11. doi:10.1007/s10803-011-1206-0

6. Meredith, A., Hussain, Z., & Griffiths, M. D. (2009). Online gaming: A scoping study of massively multi-player online role playing games. *Electronic Commerce Research, 9*(1-2), 3-26. doi:10.1007/s10660-009-9029-1

7. Ravenek, M. J., Bryson-Campbell, M. M., Shaw, L., & Hughes, I. D. (2010). Perspectives on prevention, assessment, and rehabilitation of low back pain in WORK. *Work, 35*(3), 269-282. doi:10.3233/WOR-2010-0990

8. Sawka, A. M., Ismaila, N., Cranney, A., Thabane, L., Kastner, M., Gafni, A., et al. (2010). A scoping review of strategies for the prevention of hip fracture in elderly nursing home residents. *Plos One, 5*(3) doi:10.1371/journal.pone.0009515

9. Spilsbury, K., Hewitt, C., Stirk, L., & Bowman, C. (2011). The relationship between nurse staffing and quality of care in nursing homes: A systematic review. *International Journal of Nursing Studies, 48*(6), 732-750. doi:10.1016/j.ijnurstu.2011.02.014

10. Vissandjee, B., Hyman, I., Spitzer, D. L., Apale, A., & Kamrun, N. (2007). Integration, clarification, substantiation: Sex, gender, ethnicity and migration as social determinants of women's health. *Journal of International Women's Studies, 8*(4), 32-48.

Updated search

A. Electronic databases

**Database/platform:**	**SciVerse Scopus (Elsevier)**
**Date coverage:**	1823 to present (greater than half of which are from 1996 to present)
**Library:**	Public Health Agency of Canada & Health Canada Libraries
**Date of search:**	Monday, October 1, 2012
**Limits:**	In: "Article Title, Abstract, Keywords"<br>Published: ">2010"<br>Document type: "All"<br>Subject Areas: All checked (default)
**Search query:**	"scoping study" OR "scoping review" OR "scoping project" OR "literature scoping" OR "literature mapping" OR "scoping exercise" OR "scoping report" OR "evidence map" OR "evidence mapping" OR "systematic map" OR "systematic mapping" OR "rapid review"
**Number of hits:**	329

**Database/platform:**	**MEDLINE (PubMed)**
**Time coverage:**	Generally 1946 to present
**Library:**	Free access
**Date of search:**	Monday, October 1, 2012
**Limits:**	In: "All fields"<br>Publication date: “from 2011/06/01”
**Search query:**	scoping stud* OR scoping review* OR scoping project* OR literature map* OR scoping exercise* OR scoping report* OR evidence map* OR systematic map* OR rapid review*
**Number of hits:**	158

**Database/platform:**	**CINAHL® (EBSCO)**
**Time coverage:**	Full text coverage from 1981 to present
**Library:**	University of Guelph
**Date of search:**	Monday, October 1, 2012
**Limits:**	In: "Select a field (optional)"<br>Search mode: "Boolean/Phrase"<br>Apply related words: [checked]<br>Limit your results: “Published date from 2011-06-01 to 2012-10-31”
**Search query:**	"scoping stud*" OR "scoping review*" OR "scoping project*" OR "literature map*" OR "scoping exercise*" OR "scoping report*" OR "evidence map*" OR "systematic map*" OR "rapid review*"
**Number of hits:**	75

**Database/platform:**	**Current Contents Connect® (ISI Web of Knowledge)**
**Time coverage:**	1998 to present
**Library:**	University of Guelph
**Date of search:**	Monday, October 1, 2012
**Limits:**	In: "Topic"<br>Timespan: "2011-06-01 - 2012-10-01"

	Current Contents Editions: all checked (default)<br>Current Contents Connect on WofS from 1998-
**Search query:**	"scoping study" OR "scoping studies" OR "scoping review*" OR "literature scoping" OR "literature mapping" OR "literature map" OR "scoping project*" OR "scoping exercise*" OR "evidence mapping" OR "systematic map" OR "systematic mapping" OR "rapid review"
**Number of hits:**	145

B. Web search and websites

**Website:**	**SciVerse Hub**
**Date of search:**	Monday, October 1, 2012
**Search query:**	"scoping study" OR "scoping review" OR "scoping project" OR "literature scoping" OR "literature mapping" OR "scoping exercise" OR "scoping report" OR "evidence map" OR "evidence mapping" OR "systematic map" OR "systematic mapping" OR "rapid review"
**Exact query:**	(TITLE-ABS-KEY("scoping study" OR "scoping review" OR "scoping project" OR "literature scoping" OR "literature mapping" OR "scoping exercise" OR "scoping report" OR "evidence map" OR "evidence mapping" OR "systematic map" OR "systematic mapping" OR "rapid review") AND PUBYEAR > 2009) and not srctype(jnl or pat or sc or mdc)
**Limits:**	In: "Article Title, Abstract, Keywords"<br>Published: ">2010"
**Number of hits:**	0
**Citations added:**	0

**Website:**	**National Institute for Health Research (NIHR)**<br>**Service Delivery and Organisation Programme (SDO)**<br>**Health Services and Delivery Research Programme**
**URL:**	http://www.sdo.nihr.ac.uk/newspublicationsandevents.html
**Date of search:**	Monday, October 15, 2012
**Number of hits:**	“about 775”
**Notes:**	Reviewed first 50 hits
**Citations added:**	2

**Website:**	**The University of York Social Policy Research Unit**
**URL:**	http://php.york.ac.uk/inst/spru/pubs/main.php
**Date of search:**	Monday, October 15, 2012
**Search date range:**	From 2011 to present
**Limits:**	Publication type: All<br>Research team: All<br>Subject keyword: scoping
**Citations added:**	0

**Website:**	**National Institute for Health and Care Excellence Evidence Search**
**URL:**	http://www.evidence.nhs.uk/search?q=scoping
**Date of search:**	Monday, October 15, 2012
**Search term:**	scoping
**Number of hits:**	2737
**Notes:**	Reviewed first 100 hits
**Citations added:**	35

Website:	The Department of Health
URL:	http://www.dh.gov.uk/en/Publicationsandstatistics/Publications/PublicationsLibrary/index.htm
Date of search:	Monday, October 15, 2012
Search term:	scoping
Search date range:	“1 June 2011” to “16 October 2012”
Number of hits:	0
Citations added:	0

Website:	British Association for Counselling & Psychotherapy
URL:	http://bacp.co.uk/research/publications/index.php
Date of search:	Monday, October 15, 2012
Citations added:	1

Website:	Google
URL:	google.com
Date of search:	Monday, October 15, 2012
Search query:	“scoping review”
Notes:	Reviewed first 100 hits
Citations added:	10
