# Supplementary material for: A scoping review of scoping reviews: advancing the approach and enhancing the consistency
Source: Res Synth Methods. 2014 Jul 24;5(4):371–85. doi: 10.1002/jrsm.1123 (PMC4491356; doi:10.1002/jrsm.1123)
Supplement: Supplementary file 2 — Supporting info item [file jrsm0005-0371-sd2.pdf]

Additional file 2: Title and abstract relevance screening tool

1. Does the citation report the use of a scoping review[1] methodology to identify and characterize the existing literature or evidence base on a broad topic?
   - ❒ Yes, a primary scoping review.
   - ❒ No, a methodological review of scoping reviews[2].
   - ❒ No, a narrative review[3] of scoping reviews.
   - ❒ No, none of the above.[4]
   - ❒ Can't tell.[5]

2. Does the citation describe research in English, French or Spanish[6]?
   - ❒ Yes, in English.
   - ❒ Yes, in French.
   - ❒ Yes, in Spanish.
   - ❒ No.
   - ❒ Can't tell

Reviewer Decision:
The following will be incorporated into the ScS electronic review and will happen automatically:

- If the reviewer answer is "Yes" to both questions 1 and 2, the article will be included in RS2 for further screening and appraisal.
- If the reviewer answer is "Can't tell" for either or both questions, the full article will be obtained for further appraisal and decision making on this level.

---

[1] A scoping review is a type of literature review that aims to 'map' the relevant literature in a field of interest (Arkey & O'Malley, 2005). They can be used to summarize findings of research, identify research gaps, and inform a systematic review (Arksey & O'Malley, 2005; Armstrong *et al*., 2010). Other terminologies used to describe scoping reviews may include, but is not limited to: scoping studies, evidence mapping, systematic mapping, scoping literature reviews, literature mapping, literature scoping, scoping project and rapid reviews.

[2] A methodological review would focus on the methodology of primary scoping reviews rather than on their results. It could be used to identify methodological strengths and weaknesses of published scoping reviews, and examine how research practices differ across groups, time or settings.

[3] A narrative review provides a general overview of the research literature in a specific area.

[4] Example: systematic reviews.

[5] Reviewers should only select the "Can't tell" option if the article may be relevant. If the article is obviously not relevant, "No" should be selected. Full articles must be obtained for any "Can't tell" responses.

[6] If the citation states that the article is in a language other than English, French or Spanish, "No" should be selected.
