# Supplementary material for: A scoping review of scoping reviews: advancing the approach and enhancing the consistency
Source: Res Synth Methods. 2014 Jul 24;5(4):371–85. doi: 10.1002/jrsm.1123 (PMC4491356; doi:10.1002/jrsm.1123)
Supplement: Supplementary file 3 — Supporting info item [file jrsm0005-0371-sd3.pdf]

Additional file 3: Data characterization and utility tool

**A. General Study Characteristics**		
**Variable**	**Category**	**Explanation**
1. Publication type	❒ Journal article<br>❒ Conference proceeding<br>❒ Thesis<br>❒ Government or research station report<br>❒ Other (please specify:_ _ _ _ _ _ _ _ _)	Please select one.
2. Institution(s) that funded the study	_ _ _ _ _ _ _ _ _ _ _ _ _ _ _ _ _ _ _ _<br>❒ None<br>❒ Not reported	Please list if reported.<br><br>**None**: Select if nothing is declared under the "Disclosures" (or similar) section, or it is specifically stated that the study did not receive funding.<br><br>*Include stipend and scholarship money (Sep 2 2011)
3. Institution(s) that commissioned the study	_ _ _ _ _ _ _ _ _ _ _ _ _ _ _ _ _ _ _ _<br>❒ Same as above<br>❒ None<br>❒ Not reported	Please list if reported.<br><br>**None**: Select if nothing is declared under the "Disclosures" (or similar) section, or it is specifically stated that the study was not commissioned.
4. Study sector setting	❒ Agriculture and agri-food<br>❒ Business<br>❒ Education<br>❒ Health<br>❒ Social sciences<br>❒ Other (please specify:_ _ _ _ _ _ _ _ _)	Check all that apply.<br><br>**Agriculture and agri-food**: field crops, forestry, fishery, livestock.<br>**Business**: manufacturing, commerce, finance.<br>**Education**: K-12, post-secondary, professional development.<br>**Health**: nursing, medicine, dentistry, nutrition, public health, occupational therapy.<br>Social sciences: psychology, sociology, political science, criminology, anthropology, mental health.

		Social sciences: psychology, sociology, political science, criminology, anthropology, behaviour modification.<br>**Other**: Environment, Software Technology
5. How was ScS defined in the study?	_ _ _ _ _ _ _ _ _ _ _ _ _ _ _ _ _ _ _ _<br>❒ Not defined	If defined, please copy-and-paste author(s) wording into the text box **<u>and/or</u>** list page number, column, and paragraph number.
6. Does the article report the use of a scoping review methodology to identify and characterize the existing literature or evidence base on a broad topic?	❒ Yes, a primary scoping review<br>❒ No, a methodological review of scoping reviews<br>❒ No, a narrative review of scoping reviews<br>❒ No, none of the above	Check one.
**Continue ONLY if the answer to the above question is "Yes, a primary scoping review."**		
7. What is the broad topic addressed by the ScS?	_ _ _ _ _ _ _ _ _ _ _ _ _ _ _ _ _ _ _ _	Please copy-and-paste author(s) wording into the text box **<u>and/or</u>** list page number, column, and paragraph number.
8. What was the main purpose or objective of using a ScS methodology, as stated by the author(s) in the Introduction or Methods section?	❒ To identify, characterize and summarize research evidence on a topic (including identification of research gaps)<br>❒ To identify or prioritize questions for a systematic review<br>❒ Other (please specify:_ _ _ _ _ _ _ _ _)<br>❒ Not reported	Check all that apply.

**B. Scoping Review Characterization (& Utility) Tool**		
**Variable**	**Category**	**Explanation**
9. Does the study reference a ScS framework?	❒ Yes (please specify:_ _ _ _ _ _ _ _ _)<br>❒ No	If applicable, please specify the author(s) of the framework (*e.g.*, Pham *et al.*, 2001).
10. Which of the following was reported for the search?	❒ Complete search strings or list of keywords<br>❒ Publication date range<br>❒ Search limits or parameters<br>❒ Date of search	Check all that apply.<br><br>**Complete search strings/queries**: Do not check if only subject

	❒ Date of updated search<br>❒ List of data sources<br>❒ Directed to supporting document(s) for information	headings are reported.<br>**Publication date range**: *e.g.*, 1995-2008. If date of search is reported, then “1980-present” or “published since 1980” are okay.<br>**Date of search**: Check if publication date range is reported as “1980 to June 2006” or “published up until June 2006”; can assume that date of search was ~June 2006 (unless otherwise reported).<br>**Search limits or parameters**: *e.g.*, language, geography.<br>**Directed to supporting document(s)**: *e.g.*, appendix.
11. Which data sources were included in the search strategy?	❒ Electronic bibliographic databases<br>❒ Bibliography/reference list from relevant article(s)<br>❒ Hand searching of select journal(s)<br>❒ Internet/website searching<br>❒ Consultation with experts<br>❒ Other (please specify:_ _ _ _ _ _ _ _ _)<br>❒ Directed to supporting document(s)<br>❒ Not reported	Check all that apply.<br><br>**Directed to supporting document(s)**: *e.g.*, appendix.
12. How was data/study selection performed?	❒ Relevance screening of titles and abstracts conducted by one reviewer<br>❒ Relevance screening of titles and abstracts conducted by two or more independent reviewers<br>❒ Relevance screening of full articles/papers conducted by one reviewer<br>❒ Relevance screening of full articles/papers conducted by two or more independent reviewers<br>❒ Using a relevance screening form or tool with a priori-determined inclusion and exclusion criteria	Check all that apply.<br><br>***a priori***: Determined prior to start of study selection.<br><br>**Directed to supporting document(s)**: *e.g.*, appendix.<br><br>**Using a relevance screening form or tool with a priori-determined inclusion and exclusion criteria**: Select if the use of any inclusion/exclusion criteria is reported for relevance screening.

	❒ Other<br>(please specify:_ _ _ _ _ _ _ _ _)<br>❒ Directed to supporting document(s)<br>❒ Not reported	
13. If relevance screening was carried out by more than 1 reviewer, how was the level of reviewer agreement reported?	❒ Cohen's kappa<br>❒ Percentage agreement<br>❒ Other<br>(please specify:_ _ _ _ _ _ _ _ _)<br>❒ Not reported/calculated<br>❒ Not applicable	Check one.<br><br>**Not applicable**: Select if there was only one reviewer.
14. What type(s) of primary (original) research studies were included* in the ScS?<br><br>*a study is considered "included" if it listed in a table and/or synthesized in the results section	❒ All study designs<br><br>[Otherwise, check all that apply below:]<br>❒ Observational studies<br>(if reported, please specify type(s): _ _ _ _ _ _ _ _ _ _ _ _ _)<br>❒ Experimental studies<br>(if reported, please specify type(s): _ _ _ _ _ _ _ _ _ _ _ _ _)<br>❒ Qualitative studies<br>(if reported, please specify type(s): _ _ _ _ _ _ _ _ _ _ _ _ _)<br>❒ Other<br>(please specify:_ _ _ _ _ _ _ _ _)<br>❒ Not specified	Check all that apply.<br><br>**All study designs**: Select if a list of study designs is listed in a non-specific manner.<br>**Primary (original) research**: Investigator(s) collected samples or data themselves for analysis.<br>**Observational study**: Assignment of subjects into a treated group versus a control group is outside the control of the investigator. *E.g.*: cross-sectional study, cohort study, case-control study.<br>**Experimental study**: Each subject is randomly assigned to a treated group or a control group before the start of the treatment. *E.g.*: challenge trial, controlled trial, quasi-experiments.<br>**Qualitative study**: Aimed at understanding social phenomena, exploring issues, and answering questions of "why" and "how". *E.g.*: focus groups, interviews.<br>**Not specified**: Select if "textbook" is listed without any additional information.
15. Were secondary research studies included* in the ScS?	❒ Yes<br>(if reported, please specify type(s): _ _ _ _ _ _ _ _ _ _ _ _ _)<br>❒ No	Check one.<br><br>**Secondary research**: Summary, collation and/or

*a study is considered “included” if it listed in a table and/or synthesized in the results section	❒ Not reported	synthesis of existing data. *E.g.*: systematic review, meta analysis, narrative review, scoping review.<br>**No**: Select if only articles describing primary (original) research were included.
16. What type(s) of publication(s) were included* in the ScS?<br><br>*a study is considered “included” if it listed in a table and/or synthesized in the results section	❒ All publication types<br><br>[Otherwise, check all that apply below:]<br>❒ Articles published in scientific journals (peer-reviewed and non-peer-reviewed)<br>❒ Research documents not published in scientific journals<br>❒ Thesis dissertations<br>❒ Other (please specify:_ _ _ _ _ _ _ _ _)<br>❒ Not reported	Check all that apply.<br><br>**Research documents not published in scientific journals**: *e.g.*, research studies published on a website, in a report or policy paper.
17. How was data extraction of studies conducted?	❒ By one reviewer<br>❒ By two or more independent reviewers<br>❒ Using a data extraction form or tool<br>❒ Other (please specify:_ _ _ _ _ _ _ _ _)<br>❒ Directed to supporting document(s)<br>❒ Not reported	Check all that apply.<br><br>**Directed to supporting document(s)**: *e.g.*, appendix.<br>**Other**: Any report of coding analysis.
18. Was the flow of the literature search and selection of studies through the review reported?	❒ Yes, using a flow diagram<br>❒ Yes, in the text<br>❒ Directed to supporting document(s)<br>❒ No	Check all that apply.<br><br>**Yes**: Select if numbers are reported for each step in the selection process. *E.g.*, if only 2 steps are reported, only 2 figures are required.<br>**Directed to supporting document(s)**: *e.g.*, appendix.
19. In which format(s) were the results summarized?	❒ Narrative<br>❒ Graphical form<br>❒ Tabular form<br>❒ Meta-analysis<br>❒ Other (please specify:_ _ _ _ _ _ _ _ _)	Check all that apply.
20. Was quality assessment of included studies reported?	❒ Yes<br>❒ No	Check one.

21. How many studies were included for review, as reported by the author(s)?	_ _ _ _ _ _ _ _ _ _ _ _ _ _ _ _ _ _ _ _<br>❒ Not reported	Please list the number of studies that were reported to be relevant and included in the charting process.
22. Did the author(s) report the use of specialized computer software or application(s) to map the data?	❒ Yes (please specify:_ _ _ _ _ _ _ _ _)<br>❒ No	Check one.

3. Impact on future research and policy- and/or decision-making		
**Variable**	**Category**	**Explanation**
23. How was evidence from the ScS used by the author(s)?	_ _ _ _ _ _ _ _ _ _ _ _ _ _ _ _ _ _ _ _<br>❒ Not reported	Please copy-and-paste author(s) wording into the text box **and/or** list page number, column, and paragraph number. *E.g.*: To recommend or support a policy action; to frame options for action implementation; to identify/recommend questions and topics for future research; to inform and/or frame questions for a systematic review; to inform gaps in the existing research or evidence; and to clarify a particular problem or issue.
24. Who were the primary stakeholders for the ScS, as reported by the author(s)?	❒ Researchers<br>❒ Practitioners, clinicians or service providers<br>❒ Consumers or patients<br>❒ General public<br>❒ Policy- and/or decision-makers<br>❒ Private sector or industry<br>❒ Research funding body<br>❒ Volunteer sector or non-governmental organization<br>❒ Media<br>❒ Other (please specify:_ _ _ _ _ _ _ _ _)<br>❒ Not reported	Check all that apply.<br><br>**Policy- and/or decision-makers**: In organization, community or government.<br><br>*Check if:<br>• author(s) report somesort of involvement of the stakeholder(s) in the study process<br>• study has been commissioned<br>• experts were consulted
25. What was the degree of stakeholder engagement in	❒ Shaping the research question(s)	Check all that apply.

the study process, as reported by the author(s)?	❒ Identification of relevant studies<br>❒ Interpretation of study findings<br>❒ Provision of comments at the report writing stage<br>❒ Dissemination of study results<br>❒ Moving the results into their practice<br>❒ Other (please specify:_ _ _ _ _ _ _ _ _)<br>❒ Not reported<br>❒ Not applicable	**Not applicable**: Select if involvement of stakeholder(s) was not reported by the author(s).
26. Was knowledge translation and transfer (KTT) reported as part the study process?	❒ Yes, integrated KTT<br>❒ Yes, end of grant KTT<br>❒ No	Check one.<br><br>**Knowledge translation**: A dynamic and iterative process that includes synthesis, dissemination, exchange and ethically-sound application of knowledge to improve health, provide more effective health services and products and strengthen the health care system.<br>**Integrated KTT**: Throughout the research process (*i.e.*, from idea formulation to dissemination of research results). This form of KTT is often reported in the Methods section.<br>**End of grant KTT**: Dissemination of research findings once a project is completed. This form of KTT is more often only reported in the Discussion section.
27. Were any of following KTT activities used to disseminate research findings to stakeholders?	❒ Workshop<br>❒ Document distribution<br>❒ Presentation(s)<br>❒ Other (please specify:_ _ _ _ _ _ _ _ _)<br>❒ Not reported	Check all that apply.<br><br>**Document distribution**: *e.g.*, briefing paper, technical report, summary document.<br>**Presentation(s)**: *e.g.*, conference, seminar, meeting.
28. Was an evaluation of	❒ Yes	Check one.

the effectiveness of the KTT activity (or activities) reported by the author(s)?	❒ No<br>❒ Not applicable	**No**: Select if a KTT activity was reported, but an evaluation of its effectiveness was not reported.<br>**Not applicable**: Select if a KTT activity was not reported in the study.
29. Does the author propose "next steps" or actions, based on the ScS results?	❒ KTT activities to disseminate research findings<br>❒ Implementation of a systematic review<br>❒ Additional research that is not a systematic review<br>❒ Other (please specify:_ _ _ _ _ _ _ _ _)<br>❒ Not reported	Check all that apply.
30. Was feedback regarding the overall scoping review process reported in the Discussion or Conclusion section?	❒ Yes (please list page number, column, and paragraph number: _ _ _ _ _ _ _ _ _ _ _ _ _ _)<br>❒ No	If defined, please copy-and-paste author(s) wording into the text box **<u>and/or</u>** list page number, column, and paragraph number.<br><br>**Feedback**: From either the author(s) or stakeholder(s); *e.g.*, overall length of the study process, practicality or utility, strengths or limitations reported.
31. Please provide any additional comments or notes in the space below:		
