# Supplementary material for: A scoping review of scoping reviews: advancing the approach and enhancing the consistency
Source: Res Synth Methods. 2014 Jul 24;5(4):371–85. doi: 10.1002/jrsm.1123 (PMC4491356; doi:10.1002/jrsm.1123)
Supplement: Supplementary file 4 — Supporting info item [file jrsm0005-0371-sd4.pdf]

Additional file 4: List of included scoping reviews

1. Aarts JW, van den Haak P, Nelen WL, Tuil WS, Faber MJ, Kremer JA: **Patient-focused internet interventions in reproductive medicine: a scoping review.** *Hum Reprod Update* 2012, **18**:211-227.

2. Abdul Qadoos Bilal Khan M, Shahbaz A, Shahzad F: **A systematic mapping on selection of open source software license economic and social perspective.** *J Theor Appl Inf Technol* 2011, **25**:120-125.

3. Abraham A, Sommerhalder K, Abel T: **Landscape and well-being: a scoping study on the health-promoting impact of outdoor environments.** *Int J Public Health* 2010, **55**:59-69.

4. Acuña ST, Castro JW, Dieste O and Juristo N: A systematic mapping study on the open source software development process. *In: IET Seminar Digest:* Edited by: Anonymous . 2012:42-46.

5. Adams J, White M: **Are the stages of change socioeconomically distributed? A scoping review.** *Am J Health Promot* 2007, **21**:237-247.

6. Agomo CO: **The role of community pharmacists in public health: A scoping review of the literature.** *J Pharm Health Serv Res* 2012, **3**:25-33.

7. Akpokodje J, Bowles R, Tigere E: *Evidence-based approaches to crime prevention in developing countries - A scoping review of the literature.* York: Centre for Criminal Justice Economics and Psychology, University of York; 2002.

8. Alam R, Speed S, Beaver K: **A scoping review on the experiences and preferences in accessing diabetes-related healthcare information and services by British Bangladeshis.** *Health Soc Care Community* 2012, **20**:155-171.

9. Anstee S, Price A, Young A, Barnard K, Coates B, Fraser S, Moran R: **Developing a matrix to identify and prioritise research recommendations in HIV Prevention.** *BMC Public Health* 2011, **11**:381.

10. Antonio EA, Ferrari FC and Fabbri SCPF: A systematic mapping of architectures for embedded software. *In: Proceedings - 2012 2nd Brazilian Conference on Critical Embedded Systems, CBSEC 2012:* Edited by: Anonymous . 2012:18-23.

11. Archer N, Fevrier-Thomas U, Lokker C, McKibbon KA, Straus SE: **Personal health records: a scoping review.** *J Am Med Inform Assoc* 2011, **18**:515-522.

12. Arksey H: **Scoping the field: services for carers of people with mental health problems.** *Health Soc Care Community* 2003, **11**:335-344.

13. Arksey H, Corden A, Glendinning C, Hirst M: *Minding the Money: Carers and the Managements of Financial Assets in Late Life: report of a scoping study.* York: Social Policy Research Unit, University of York; 2006.

14. Arksey H, O'Malley L, Baldwin S, Harris J: *Services to Support Carers of People with Mental Health Problems: Overview Report for the National Co-ordinating Centre for NHS Service Delivery and Organisation R & D (NCCSDO).* Southampton: National Co-ordinating Centre for NHS Service Delivery and Organisation; 2002.

15. Arshad A and Usman M: Security at software architecture level: A systematic mapping study. *In: IET Seminar Digest:* Edited by: Anonymous . 2011:164-168.

16. Attree P, French B, Milton B, Povall S, Whitehead M, Popay J: **The experience of community engagement for individuals: a rapid review of evidence.** *Health Soc Care Community* 2011, **19**:250-260.

17. Babinec PM, Rock MJ, Lorenzetti DL, Johnson JA: **Do researchers use pharmacists' communication as an outcome measure? A scoping review of pharmacist involvement in diabetes care.** *Int J Pharm Pract* 2010, **18**:183-193.

18. Bagaria J, Heggie C, Abrahams J, Murray V: **Evacuation and sheltering of hospitals in emergencies: a review of international experience.** *Prehosp Disaster Med* 2009, **24**:461-467.

19. Bagnall AM, Wilby J, Glanville J, Sowden A: *Scoping review of sabotage and/or tampering in the NHS.* York: University of York; 2004.

20. Bambra C, Joyce KE, Bellis MA, Greatley A, Greengross S, Hughes S, Lincoln P, Lobstein T, Naylor C, Salay R, Wiseman M, Maryon-Davis A: **Reducing health inequalities in priority public health conditions: using rapid review to develop proposals for evidence-based policy.** *J PUBLIC HEALTH* 2010, **32**:496-505.

21. Bamm EL, Rosenbaum P, Wilkins S: **Is Health Related Quality Of Life of people living with chronic conditions related to patient satisfaction with care?** *Disabil Rehabil* 2012, .

22. Barmi ZA, Ebrahimi AH and Feldt R: Alignment of requirements specification and testing: A systematic mapping study. *In: Proceedings - 4th IEEE International Conference on Software Testing, Verification, and Validation Workshops, ICSTW 2011:* Edited by: Anonymous . 2011:476-485.

23. Barney S, Petersen K, Svahnberg M, Aurum A, Barney H: **Software quality trade-offs: A systematic map.** *Inf Software Technol* 2012, **54**:651-662.

24. Barreiros E, Almeida A, Saraiva J and Soares S: A systematic mapping study on software engineering testbeds. *In: Proceedings - 2011 5th International Symposium on Empirical Software Engineering and Measurement, ESEM 2011:* Edited by: Anonymous . 2011:107-116.

25. Bassi J, Lau F, Bardal S: **Use of information technology in medication reconciliation: A scoping review.** *Ann Pharmacother* 2010, **44**:885-897.

26. Bates S, Coren E: *Systematic map report 1: The extent and impact of parental mental health problems on families and the acceptability, accessibility and effectiveness of interventions.* London: Social Care Institute for Excellence; 2006.

27. Baxter K, Glendinning C, Clarke S: **Making informed choices in social care: The importance of accessible information.** *Health Soc Care Community* 2008, **16**:197-207.

28. Baxter K, Glendinning C, Clarke S: *Scoping Review on Access to Information about Social Care Services.* York: Social Policy Research Unit, University of York; 2006.

29. Bell CA, Duncan G, Saini B: **Knowledge, attitudes and practices of private sector providers of tuberculosis care: a scoping review.** *Int J Tuberc Lung Dis* 2011, .

30. Bennett J, Hogarth S, Lubben F, Campbell B, Robinson A: **Talking science: The research evidence on the use of small group discussions in science teaching.** *Int J Sci Educ* 2010, **32**:69-95.

31. Bennett KM, Scornaiencki JM, Brzozowski J, Denis S, Magalhaes L: **Immigration and its Impact on Daily Occupations: A Scoping Review.** *Occup Ther Int* 2012, .

32. Bielby G, Chamberlain T, Morris M, O'Donnell L, Sharp C: *Improving the wellbeing of disabled children and young people through improving access to positive activities.* London: Centre for Excellence and Outcomes in Children and Young People's Services (C4EO); 2009.

33. Bisht R, Pitchforth E, Murray SF: **Understanding India, globalisation and health care systems: a mapping of research in the social sciences.** *Global Health* 2012, **8**:32.

34. Bjarkoy M, Turner J: *What services and skills should be part of an emergency medical services (EMS)/pre-hospital care system that can manage high demand and varied case-mix?* London: ; 2010.

35. Boeltzig H, Pilling D, Timmons JC, Johnson R: **Disability specialist staff in US One-Stop Career centers and British Jobcentre plus offices: Roles, responsibilities, and evidence of their effectiveness.** *J Disabil Policy Stud* 2010, **21**:101-115.

36. Borkhoff CM, Wieland ML, Myasoedova E, Ahmad Z, Welch V, Hawker GA, Li LC, Buchbinder R, Ueffing E, Beaton D, Cardiel MH, Gabriel SE, Guillemin F, Adebajo AO, Bombardier C, Hajjaj-Hassouni N, Tugwell P: **Reaching those most in need: a scoping review of interventions to improve health care quality for disadvantaged populations with osteoarthritis.** *Arthritis Care Res (Hoboken)* 2011, **63**:39-52.

37. Borthwick R, Newbronner L, Stuttard L: **'Out of hospital': A scoping study of services for carers of people being discharged from hospital.** *Health Soc Care Community* 2009, **17**:335-349.

38. Bostock L, Brodie I, Clapton J, Fish S, Morris M, Kearney P, Rutter D: *Increasing the number of care leavers in 'settled, safe accommodation': scoping review 3.* London: Centre for Excellence and Outcomes in Children and Young People's Services; 2009.

39. Bostrom AM, Slaughter SE, Chojecki D, Estabrooks CA: **What Do We Know About Knowledge Translation in the Care of Older Adults? A Scoping Review.** *J Am Med Dir Assoc* 2011, **13**:210-219.

40. Bottorff JL, Oliffe JL, Robinson CA, Carey J: **Gender relations and health research: a review of current practices.** *Int J Equity Health* 2011, **10**:60-60.

41. Boyd A, Chambers N, French S, King R, Shaw D, Whitehead A: *A scoping study of emergency planning and management in health care: What further research is needed?* Southampton: National Institute for Health Research Service Delivery and Organisation Programme; 2012.

42. Boydell KM, Gladstone BM, Volpe T, Allemang B, Stasiulis E: **The Production and Dissemination of Knowledge: A Scoping Review of Arts-Based Health Research.** *Forum: Qualitative Social Research* 2012, **13**.

43. Bragge P, Clavisi O, Turner T, Tavender E, Collie A, Gruen RL: **The Global Evidence Mapping Initiative: Scoping research in broad topic areas.** *BMC Med Res Methodol* 2011, **11**:92-92.

44. Branas P, Jordan R, Fry-Smith A, Burls A, Hyde C: **Treatments for fatigue in multiple sclerosis: a rapid and systematic review.** *Health Technol Assess* 2000, **4**:1-61.

45. Brearley SG, Stamataki Z, Addington-Hall J, Foster C, Hodges L, Jarrett N, Richardson A, Scott I, Sharpe M, Stark D, Siller C, Ziegler L, Amir Z: **The physical and practical problems experienced by cancer survivors: A rapid review and synthesis of the literature.** *Eur J Oncol Nurs* 2011, **15**:204-212.

46. Brien SE, Lorenzetti DL, Lewis S, Kennedy J, Ghali WA: **Overview of a formal scoping review on health system report cards.** *Implement Sci* 2010, **5**:2.

47. Brodie I, Bostock L, Clapton J, Fish S, Fisher M, Morris M, Kearney P, Rutter D: *Improving educational outcomes for looked-after children and young people: scoping review 1.* London: Centre for Excellence and Outcomes in Children and Young People's Services; 2009.

48. Bulkeley H, Castán Broto V: **Government by experiment? Global cities and the governing of climate change.** *Trans Inst Br Geogr* 2012, .

49. Cahill J, Barkham M, Hardy G, Gilbody S, Richards D, Bower P, Audin K, Connell J: **A review and critical appraisal of measures of therapist–patient interactions in mental health settings.** *Health Technol Assess* 2008, **12**:1-86.

50. Callahan P, Liu P, Purcell R, Parker AG, Hetrick SE: **Evidence map of prevention and treatment interventions for depression in young people.** *Depress Res Treat* 2012, **2012**:820735.

51. Cameron JI, Tsoi C, Marsella A: **Optimizing stroke systems of care by enhancing transitions across care environments.** *Stroke* 2008, **39**:2637-2643.

52. Campana A, Lapointe L: **The structural "Root" causes of non-suicide terrorism: A systematic scoping review.** *Terrorism Polit Violence* 2012, **24**:79-104.

53. Campbell C, Parent M, Plangger K, Fulgoni GM: **Instant Innovation: From Zero to Full Speed in Fifteen Years How Online Offerings Have Reshaped Marketing Research.** *J Advert Res* 2011, **51**:72-86.

54. Canadian Population Health Initiative: *Urban Physical Environments and Health Inequalities: A Scoping Review of Interventions.* Ottawa: Canadian Institute for Health Information; 2012.

55. Caraher M, Dowler E: **Food projects in London: Lessons for policy and practice - A hidden sector and the need for 'more unhealthy puddings ... sometimes'.** *Health Educ J* 2007, **66**:188-205.

56. Carr-Hille R, Currie L, Dixon P: *Skill Mix in Secondary Care: A scoping exercise.* Southampton: National Co-ordinating Centre for NHS Service Delivery and Organisation; 2003.

57. Carrier A, Levasseur M, Bédard D, Desrosiers J: **Community occupational therapists' clinical reasoning: Identifying tacit knowledge.** *Aust Occup Ther J* 2010, **57**:356-365.

58. Castro Llanos JW, Acuña Castillo ST: Differences between traditional and open source development activities. In *Lecture Notes in Computer Science (including subseries Lecture Notes in Artificial Intelligence and Lecture Notes in Bioinformatics).* Edited by Anonymous 2012:131-144. [Anonymous , *vol 7343 LNCS.*

59. Catal C, Mishra D: **Test case prioritization: a systematic mapping study.** *Softw Qual J* 2012, :1-34.

60. Catts SV, O'Toole BI, Carr VJ, Lewin T, Neil A, Harris MG, Frost ADJ, Crissman BR, Eadie K, Evans RW: **Appraising evidence for intervention effectiveness in early psychosis: Conceptual framework and review of evaluation approaches.** *Aust New Zealand J Psychiatry* 2010, **44**:195-219.

61. Challen K, Goodacre SW: **Predictive scoring in non-trauma emergency patients: A scoping review.** *Emerg Med J* 2011, .

62. Challen K, Lee AC, Booth A, Gardois P, Woods HB, Goodacre SW: **Where is the evidence for emergency planning: a scoping review.** *BMC Public Health* 2012, **12**:542.

63. Chambers D, Wilson PM, Thompson CA, Hanbury A, Farley K, Light K: **Maximizing the impact of systematic reviews in health care decision making: A systematic scoping review of knowledge-translation resources.** *Milbank Q* 2011, **89**:131-156.

64. Chambers D, Wilson P, Thompson C, Harden M: **Social Network Analysis in Healthcare Settings: A Systematic Scoping Review.** *PLoS One* 2012, **7**:e41911-e41911.

65. Chung M, Dahabreh IJ, Hadar N, Ratichek SJ, Gaylor JM, Trikalinos TA, Lau J: 2011, .

66. Churchill P, Otal D, Pemberton J, Ali A, Flageole H, Walton JM: **Sclerotherapy for lymphatic malformations in children: A scoping review.** *J Pediatr Surg* 2011, **46**:912-922.

67. Clarkson PJ, Buckle P, Coleman R, Stubbs D, Ward J, Jarrett J, Lane R, Bound J: **Design for patient safety: A review of the effectiveness of design in the UK health service.** *J Eng Des* 2004, **15**:123-140.

68. Coad JE, Shaw KL: **Is children's choice in health care rhetoric or reality? A scoping review.** *J Adv Nurs* 2008, **64**:318-327.

69. Coast E, Leone T, Hirose A, Jones E: **Poverty and postnatal depression: a systematic mapping of the evidence from low and lower middle income countries.** *Health Place* 2012, **18**:1188-1197.

70. Coghlan M, Sanders D, Morris M, O'Donnell L, Benefield P, Harper A, Sharp C: *Narrowing the gap in outcomes for children from the most excluded families through inclusive practice in early years settings.* Great Britain: Centre for Excellence and Outcomes in Children and Young People’s Services (C4EO); 2008.

71. Coleman P: *Patient priorities and decision making when using emergency medical services (EMS) and the effectiveness of publicity campaigns in influencing patient behaviour.* London: Department of Health Policy Research Programme; 2010.

72. Coleman P: *Managing change resulting from service re-organisation, service development and working across service boundaries in emergency medical services (EMS).* London: Department of Health Policy Research Programme; 2010.

73. Colquhoun HL, Letts LJ, Law MC, MacDermid JC, Missiuna CA: **A scoping review of the use of theory in studies of knowledge translation.** *Can J Occup Ther* 2010, **77**:270-279.

74. Condori-Fernandez N, Daneva M, Sikkel K, Wieringa R, Dieste O and Pastor O: A systematic mapping study on empirical evaluation of software requirements specifications techniques. *In: 2009 3rd International Symposium on Empirical Software Engineering and Measurement, ESEM 2009: 15 October 2009 through 16 October 2009; Lake Buena Vista, FL.* Edited by: Anonymous . 2009:502-505.

75. Connor H, MacFarlane K: *Work Related Learning (WRL) in Higher Education—a Scoping Study.* Glasgow, Scotland: Centre for Research Lifelong Learning (CRRL), Glasgow Caledonian University; 2006.

76. Craig D, G.: **Current Occupational Therapy Publications in Home Health: A Scoping Review.** *Am J Occup Ther* 2012, **66**:338-347.

77. Crawford F: **Clinical trials in dental primary care: what research methods have been used to produce reliable evidence?** *Br Dent J* 2005, **199**:155-60; discussion 152; quiz 174.

78. Crawford JO, Laiou E: **Conservative treatment of work-related upper limb disorders - A review.** *Occup Med* 2007, **57**:4-17.

79. Cree VE, Sidhva D: **Children and HIV in Scotland: Findings from a cross-sector needs assessment of children and young people infected and affected by HIV in Scotland.** *Br J Soc Work* 2011, **41**:1586-1603.

80. Crilly T, Jashapara A, Ferlie E: *Research Utilisation & Knowledge Mobilisation: A Scoping Review of the Literature.* Southampton: National Institute for Health Research Service Delivery and Organisation Programme; 2010.

81. Cronin De Chavez A, Backett-Milburn K, Parry O, Platt S: **Understanding and researching wellbeing: Its usage in different disciplines and potential for health research and health promotion.** *Health Educ J* 2005, **64**:70-87.

82. Crooks VA, Kingsbury P, Snyder J, Johnston R: **What is known about the patient's experience of medical tourism? A scoping review.** *BMC Health Serv Res* 2010, **10**:266.

83. Curran C, Burchardt T, Knapp M, McDaid D, Li B: **Challenges in multidisciplinary systematic reviewing: A study on social exclusion and mental health policy.** *Soc Policy Adm* 2007, **41**:289-312.

84. D Piette,,,John, Lun K, Lincoln, SF Fraser,,,Hamish, N Mechael,,,Patricia, Powell J, R Khoja,,,Shariq: **Impacts of e-health on the outcomes of care in low- and middle-income countries: where do we go from here?** *Bull World Health Organ* 2012, **90**:365-372.

85. Da Mota Silveira Neto PA, Carmo MacHado ID, McGregor JD, De Almeida ES, De Lemos Meira SR: **A systematic mapping study of software product lines testing.** *Inf Software Technol* 2011, **53**:407-423.

86. Da Silva FQB, Prikladinicki R, França ACC, Monteiro CVF, Costa C, Rocha R: **An evidence-based model of distributed software development project management: Results from a systematic mapping study.** *J Software Maint Evol* .

87. da Silva FQB, Suassuna M, França ACC, Grubb AM, Gouveia TB, Monteiro CVF, dos Santos IE: **Replication of empirical studies in software engineering research: a systematic mapping study.** *Empir Software Eng* 2012, :1-57.

88. Da Silva IF, Da Mota Silveira Neto PA, O'Leary P, De Almeida ES, De Lemos Meira SR: **Agile software product lines: A systematic mapping study.** *Software Pract Exper* 2011, **41**:899-920.

89. Davis KJ, Kumar D, Wake MC: **Pelvic floor dysfunction: A scoping study exploring current service provision in the UK, interprofessional collaboration and future management priorities.** *Int J Clin Pract* 2010, **64**:1661-1670.

90. Decaria JE, Sharp C, Petrella RJ: **Scoping review report: obesity in older adults.** *Int J Obes (Lond)* 2012, **36**:1141-1150.

91. Denning D, W., Pleuvry A, Cole D, C.: **Global burden of chronic pulmonary aspergillosis as a sequel to pulmonary tuberculosis.** *Bull World Health Organ* 2011, **89**:864-872.

92. Deshpande A, Khoja S, Lorca J, McKibbon A, Rizo C, Husereau D, Jadad AR: **Asynchronous telehealth: A scoping review of analytic studies.** *Open Med* 2009, **3**:39-61.

93. DiCenso A, Martin-Misener R, Bryant-Lukosius D, Bourgeault I, Kilpatrick K, Donald F, Kaasalainen S, Harbman P, Carter N, Kioke S, Abelson J, McKinlay RJ, Pasic D, Wasyluk B, Vohra J, Charbonneau-Smith R: **Advanced practice nursing in Canada: overview of a decision support synthesis.** *Nurs Leadersh (Tor Ont)* 2010, **23**:15-34.

94. Dowrick C, Gask L, Edwards S, Aseem S, Bower P, Burroughs H, Catlin A, Chew-Graham C, Clarke P, Gabbay M, Gowers S, Hibbert D, Kovandzic M, Lamb J, Lovell K, Rogers A, Lloyd-Williams M, Waheed W: **Researching the mental health needs of hard-to-reach groups: Managing multiple sources of evidence.** *BMC Health Serv Res* 2009, **9**.

95. Drager KDR, Reichle J, Pinkoski C: **Synthesized speech output and children: A scoping review.** *Am J Speech-Lang Pathol* 2010, **19**:259-273.

96. Dryden R, Williams B, McCowan C, Themessl-Huber M: **What do we know about who does and does not attend general health checks? Findings from a narrative scoping review.** *BMC Public Health* 2012, **12**:723.

97. Durelli VHS, Araujo RF, Silva MAG, Oliveira RAP, Maldonado JC and Delamaro ME: What a long, strange trip it's been: Past, present, and future perspectives on software testing research. *In: Proceedings - 25th Brazilian Symposium on Software Engineering, SBES 2011:* Edited by: Anonymous . 2011:30-39.

98. Durelli VHS, Felizardo KR and Delamaro ME: A systematic mapping study on high-level language virtual machines. *In: 4th Workshop on Virtual Machines and Intermediate Languages, VMIL 2010, Held at the 1st ACM SIGPLAN Conference on Systems, Programming, Languages, and Applications: Software for Humanity, SPLASH 2010: 17 October 2010 through 21 October 2010; Reno, NV.* Edited by: Anonymous . 2011:

99. Elberzhager F, Münch J, Nha VTN: **A systematic mapping study on the combination of static and dynamic quality assurance techniques.** *Inf Software Technol* 2012, **54**:1-15.

100. Elberzhager F, Rosbach A, Münch J, Eschbach R: **Reducing test effort: A systematic mapping study on existing approaches.** *Inf Software Technol* 2012, **54**:1092-1106.

101. Ellen ME, Lavis JN, Ouimet M, Grimshaw J, Bedard PO: **Determining research knowledge infrastructure for healthcare systems: A qualitative study.** *Implement Sci* 2011, **6**:60.

102. Elliott R, Napper M: *The Impact of Local Labour Market Factors on the Organisation and Delivery of Health Services.* London: NCC SDO (SDO/52/2003); 2003.

103. Engström E, Runeson P: **Software product line testing - A systematic mapping study.** *Inf Software Technol* 2011, **53**:2-13.

104. Farrar AM: The application of research synthesis methods for evaluating primary research on Salmonella in broiler chickens. *University of Guelph (Canada).* University of Guelph (Canada), Canada; 2009

105. Faulkner GE, Grootendorst P, Nguyen VH, Andreyeva T, Arbour-Nicitopoulos K, Auld MC, Cash SB, Cawley J, Donnelly P, Drewnowski A, Dube L, Ferrence R, Janssen I, Lafrance J, Lakdawalla D, Mendelsen R, Powell LM, Traill WB, Windmeijer F: **Economic instruments for obesity prevention: results of a scoping review and modified Delphi survey.** *Int J Behav Nutr Phys Act* 2011, **8**:109.

106. Fayter D, Corbett M, Heirs M, Fox D, Eastwood A: **A systematic review of photodynamic therapy in the treatment of precancerous skin conditions, Barrett's oesophagus and cancers of the biliary tract, brain, head and neck, lung, oesophagus and skin.** *Health Technol Assess* 2010, **14**:3-129.

107. Feehan LM, Beck CA, Harris SR, MacIntyre DL, Li LC: **Exercise prescription after fragility fracture in older adults: A scoping review.** *Osteoporosis Int* 2011, **22**:1289-1322.

108. Felizardo KR, Macdonell SG, Mendes E, Maldonado JC: **A systematic mapping on the use of visual data mining to support the conduct of systematic literature reviews.** *J Softw* 2012, **7**:450-461.

109. Fernandez A, Insfran E, Abrahão S: **Usability evaluation methods for the web: A systematic mapping study.** *Inf Software Technol* 2011, **53**:789-817.

110. Ferreira Bastos J, Anselmo Da Mota Silveira Neto,P., Santana De Almeida E and Romero De Lemos Meira S: Adopting software product lines: A systematic mapping study. *In: IET Seminar Digest:* Edited by: Anonymous . 2011:11-20.

111. Flowerdew L, Brown R, Vincent C, Woloshynowych M: **Identifying Nontechnical Skills Associated With Safety in the Emergency Department: A Scoping Review of the Literature.** *Ann Emerg Med* 2012, **59**:386-394.

112. Forbes A, Hughes J, Ismail K, While A: *The organisation & delivery of diabetes services in the UK: a scoping exercise.* London: Queen's Printer and Controller of HMSO; 2010.

113. Forsman H, Vinnerljung B: **Interventions aiming to improve school achievements of children in out-of-home care: A scoping review.** *Child Youth Serv Rev* 2012, **34**:1084-1091.

114. Foster C, Amir Z, Jarrett N, Stamataki Z, Brearley SG, Scott I: *Scoping of Research Evidence Regarding the Health and Well-Being of Cancer Survivors: Psychological and Social Problems Faced by Cancer Survivors, and their Physical and Practical Well-Being. Technical Report.* United Kingdom: University of Southampton and University of Manchester; 2009.

115. Fotaki M, Boyd A, Smith E, McDonald R, Roland M, Sheaff R, Edwards A, Elwyn G: *Patient choice and the organisation and delivery of health services: Scoping review.* Southampton: National Co-ordinating Centre for NHS Service Delivery and Organisation (NCCSDO); 2005.

116. Freeman G, Shepperd S, Robinson I, Ehrich K, Richards S: *Continuity of Care: Report of a Scoping Exercise.* Southampton: National Co-ordinating Centre for NHS Service Delivery and Organisation R & D (NCCSDO); 2000.

117. Gagliardi AR: **"Physician Self-Audit: A Scoping Review" from the Journal of Continuing Education in the Health Professions (vol 31, pg 258, 2011).** *J Contin Educ Health Prof* 2012, **32**:149-149.

118. Gagliardi AR, Fenech D, Eskicioglu C, Nathens AB, McLeod R: **Factors influencing antibiotic prophylaxis for surgical site infection prevention in general surgery: A review of the literature.** *Can J Surg* 2009, **52**:481-489.

119. Galloway T, Blackett H, Chatwood S, Jeppesen C, Kandola K, Linton J, Bjerregaard P: **Obesity studies in the circumpolar Inuit: a scoping review.** *Int J Circumpolar Health* 2012, **71**:18698.

120. Ganann R, Fitzpatrick-Lewis D, Ciliska D, Peirson L: **Community-based interventions for enhancing access to or consumption of fruit and vegetables among five to 18-year olds: a scoping review.** *BMC Public Health* 2012, **12**:711.

121. Gangapersad J, Brouwer A, Kurilsky S, Willis E, Shaw L: **A scoping review of the knowledge base in WORK that addresses work related outcomes for individuals with chronic pain.** *Work* 2010, **35**:283-299.

122. Gardois P, Colombi N, Grillo G, Villanacci M, C.: **Implementation of Web 2.0 services in academic, medical and research libraries: a scoping review.** *HEALTH INF LIBR J* 2012, **29**:90-109.

123. Gentles SJ, Lokker C, McKibbon KA: **Health information technology to facilitate communication involving health care providers, caregivers, and pediatric patients: A scoping review.** *J Med Internet Res* 2010, **12**:e22.

124. Goldner E, M., Jeffries V, Bilsker D, Jenkins E, Menear M, Petermann L: **Knowledge translation in mental health: a scoping review.** *HEALTHC POLICY* 2011, **7**:83-98.

125. Gomes JS, Neto PADMS, Cruzes DS and Almeida ESD: 25 Years of software engineering in Brazil: An analysis of SBES history. *In: Proceedings - 25th Brazilian Symposium on Software Engineering, SBES 2011:* Edited by: Anonymous . 2011:4-13.

126. Goodridge D, Hawranik P, Duncan V, Turner H: **Socioeconomic disparities in home health care service access and utilization: A scoping review.** *Int J Nurs Stud* 2012, .

127. Gordon AL, Logan PA, Jones RG, Forrester-Paton C, Mamo JP, Gladman JR: **A systematic mapping review of Randomized Controlled Trials (RCTs) in care homes.** *BMC Geriatr* 2012, **12**:31.

128. Goryakin Y, Griffiths P, Maben J: **Economic evaluation of nurse staffing and nurse substitution in health care: A scoping review.** *Int J Nurs Stud* 2011, **48**:501-512.

129. Gowing LR, Ali RL, White JM: **Systematic review processes and the management of opioid withdrawal.** *Aust New Zealand J Public Health* 2000, **24**:427-431.

130. Gray L, Ng H, Bartlett D: **The gross motor function classification system: An update on impact and clinical utility.** *Pediatr Phys Ther* 2010, **22**:315-320.

131. Greer N, Brasure M, Wilt TJ: 2012, .

132. Greyson DL, Becu ARE, Morgan SG: **Sex, drugs and gender roles: Mapping the use of sex and gender based analysis in pharmaceutical policy research.** *Int J Equity Health* 2010, **9**.

133. Gridley K: Good social care for people with complex needs. A scoping study (review). *In: Getting it Right when it's Complex: Good Social Care for People with Severe and Complex Needs, Making Research Count Conference, University of York, York, 11 July 2012:* Edited by: Anonymous . 2012:

134. Griffiths P, Bennett J, Smith E: **The size, extent and nature of the learning disability nursing research base: A systematic scoping review.** *Int J Nurs Stud* 2009, **46**:490-507.

135. Griffiths P, Bennett J, Smith E: *The research base for learning disability nursing: A rapid scoping review.* London: Nursing Research Unit Kings College London; 2007.

136. Griffiths P, Renz A, Hughes J, Rafferty AM: **Impact of organisation and management factors on infection control in hospitals: a scoping review.** *J Hosp Infect* 2009, **73**:1-14.

137. Griffiths P, Richardson A, Blackwell R: **Outcomes sensitive to nursing service quality in ambulatory cancer chemotherapy: Systematic scoping review.** *EUR J ONCOL NURS* 2012, **16**:238-246.

138. Guilcher SJ, Craven BC, McColl MA, Lemieux-Charles L, Casciaro T, Jaglal SB: **Application of the Andersen's health care utilization framework to secondary complications of spinal cord injury: a scoping review.** *Disabil Rehabil* 2012, **34**:531-541.

139. Gulliford M, Morgan M, Hughes D, Beech R, Figeroa-Munoz J, Gibson B, Hudson M, Arumugam C, Connell P, Mohiddin A, Sedgwick J: *Access to Health Care: Report of a Scoping Exercise.* Southampton: National Co-ordinating Centre for NHS Service Delivery and Organisation R & D (NCCSDO); 2001.

140. Gysels M, Evans N, Menaca A, Andrew E, Toscani F, Finetti S, Pasman HR, Higginson I, Harding R, Pool R, Project PRISMA: **Culture and end of life care: a scoping exercise in seven European countries.** *PLoS One* 2012, **7**:e34188.

141. Gysels M, Higginson I, White P, Barclay S, Worth A, Murray S, Shipman C, Dewar S, Richardson A, Hotopf M, Lorenz K, Koffman J: *A Scoping Exercise on Generalist Services for Adults at the End of Life: Research, Knowledge, Policy and Future Research Needs.* National Co-ordinating Centre for NHS Service Delivery and Organisation R & D (NCCSDO); 2007.

142. Haaz S, Bartlett SJ: **Yoga for Arthritis: A Scoping Review.** *Rheum Dis Clin North Am* 2011, **37**:33-46.

143. Hagell A, Dowling SD: *Scoping Review of Literature on the Health and Care of Mentally Disordered Offenders.* ; 1999.

144. Hand C, Law M, C., McColl M, Ann, Hanna S, Elliott S, J.: **Neighborhood Influences on Participation Among Older Adults With Chronic Health Conditions: A Scoping Review.** *OTJR OCCUP PARTICIPATION HEALTH* 2012, **32**:95-103.

145. Hand C, Law M, McColl M, Ann: **Occupational Therapy Interventions for Chronic Diseases: A Scoping Review.** *Am J Occup Ther* 2011, **65**:428-436.

146. Hand C, Letts L: *Occupational Therapy Research and Practice involving Adults with Chronic Diseases: A Scoping Review and Internet Scan.* Ottawa: Canadian Association of Occupational Therapists; 2009.

147. Harden A, Kavanagh J, Powell C, Oliver K, Oakley A: *A scoping review of the evidence relevant to life checks for young people aged 9 to 14 years.* London: EPPI-Centre, Social Science Research Unit, Institute of Education, University of London; 2007.

148. Harris J, Kroll T, Law J, Bolik F: *Disability and Homelessness in Central and Northern Scotland.* Dundee: Interdisciplinary Disability Research Institute; 2006.

149. Hartman L, R., Magalhães L, Mandich A: **What Does Parental Divorce or Marital Separation Mean for Adolescents? A Scoping Review of North American Literature.** *J DIVORCE REMARRIAGE* 2011, **52**:490-518.

150. Hazel N: **Holidays for children and families in need: An exploration of the research and policy context for social tourism in the UK.** *Child Soc* 2005, **19**:225-236.

151. Healthcare Improvement Scotland: *In patients with severe medically refractory gastroparesis (such as those requiring nutritional support), how effective and cost effective is gastric electrical stimulation (EnterraTM device) in reducing symptoms, reducing requirement for nutritional support or hospitalisation and improving quality of life, when compared with medical or alternative surgical management?* Edingburgh: Healthcare Improvement Scotland; 2012.

152. Healthcare Improvement Scotland: *What is the relative clinical effectiveness, cost effectiveness and safety of different bariatric surgery techniques (gastric bypass, gastric banding and sleeve gastrectomy)?* Edinburgh: Healthcare Improvement Scotland; 2012.

153. Healthcare Improvement Scotland: *In radiotherapy for cancer, what are the patient safety benefits and resource implications of the various in vivo methods of dosimetry to check received radiation dose, compared with pretreatment verification only?* Edinburgh: Healthcare Improvement Scotland; 2012.

154. Healthcare Improvement Scotland: *Is it clinically and cost effective to perform second-eye cataract surgery in the absence of other ocular co-morbidities in patients who have already had first-eye surgery?* Edinburgh: Healthcare Improvement Scotland; 2012.

155. Healthcare Improvement Scotland: *What is the impact of using thresholds (both for referral and surgery) for first-eye cataract surgery on the delivery of the cataract service and the resources associated with it?* Edinburgh: Healthcare Improvement Scotland; 2012.

156. Healthcare Improvement Scotland: *The clinical and cost-effectiveness of radiofrequency ablation for lung cancer.* Edinburgh: Healthcare Improvement Scotland; 2012.

157. Healthcare Improvement Scotland: *Is there a difference in operative mortality between endovascular aneurysm repair and open surgery in elective abdominal aortic aneurysm?* Edinburgh: Healthcare Improvement Scotland; 2011.

158. Healthcare Improvement Scotland: *What is the published evidence of an association between hospital volume and operative mortality for surgical repair (open and endovascular) of unruptured and ruptured abdominal aortic aneurysms?* Edinburgh: Healthcare Improvement Scotland; 2011.

159. Healthcare Improvement Scotland: *What is the published evidence of an association between hospital volume and outcome in elective carotid endarterectomy surgery?* Edinburgh: Healthcare Improvement Scotland; 2011.

160. Healthcare Improvement Scotland: *What implications does the organisation of vascular services have for rates of amputation?* Edinburgh: Healthcare Improvement Scotland; 2011.

161. Heller T, McCubbin JA, Drum C, Peterson J: **Physical activity and nutrition health promotion interventions: What is working for people with intellectual disabilities?** *Intellect Dev Disabil* 2011, **49**:26-36.

162. Hempel S, Chambers D, Bagnall A-, Forbes C: **Risk factors for chronic fatigue syndrome/myalgic encephalomyelitis: A systematic scoping review of multiple predictor studies.** *Psychol Med* 2008, **38**:915-926.

163. Hempel S, Norman G, Golder S, Aguiar-Ibáñez R, Eastwood A: **Psychosocial interventions for non-professional carers of people with Parkinson's disease: A systematic scoping review.** *J Adv Nurs* 2008, **64**:214-228.

164. Hewitt C, Lankshear A, Kazanjian A, Maynard A, Sheldon T, Smith K: *Health Service Workforce and Health Outcomes: A Scoping Study.* York: National Co-ordinating Centre for NHS Service Delivery and Organisation R & D (NCCSDO); 2005.

165. Hitch D: **Better access to mental health: Mapping the evidence supporting participation in meaningful occupations.** *Adv Ment Health* 2012, **10**:181-189.

166. Hooker L, Ward B, Verrinder G: **Domestic violence screening in Maternal & Child Health nursing practice: a scoping review.** *Contemp Nurse* 2012, .

167. Horne R, Weinman J, Barber N, Elliott R, Morgan M: **Concordance, Adherence and Compliance in Medicine Taking.** 2005, :1-312.

168. Hosking J, Campbell-Lendrum D: **How Well Does Climate Change and Human Health Research Match the Demands of Policymakers? A Scoping Review.** *Environ Health Perspect* 2012, **120**:1076-1082.

169. Hundley VA, Avan BI, Braunholtz D, Fitzmaurice AE, Graham WJ: **Lessons regarding the use of birth kits in low resource countries.** *Midwifery* 2010, .

170. Hunter KF, Wagg A, Kerridge T, Chick H, Chambers T: **Falls risk reduction and treatment of overactive bladder symptoms with antimuscarinic agents: A scoping review.** *Neurourol Urodyn* 2011, **30**:490-494.

171. Hussein S, Manthorpe J, Stevens M: **People in places: A qualitative exploration of recruitment agencies' perspectives on the employment of international social workers in the UK.** *Br J Soc Work* 2010, **40**:1000-1016.

172. Ilic S, Rajić A, Britton CJ, Grasso E, Wilkins W, Totton S, Wilhelm B, Waddell L, LeJeune JT: **A scoping study characterizing prevalence, risk factor and intervention research, published between 1990 and 2010, for microbial hazards in leafy green vegetables.** *Food Control* 2012, **23**:7-19.

173. Istomina N, Suominen T, Razbadauskas A, Leino-Kilpi H: **Research on the quality of abdominal surgical nursing care: A scoping review.** *Medicina (Croatia)* 2011, **47**:245-256.

174. Jalali S and Wohlin C: Agile practices in global software engineering - A systematic map. *In: 5th International Conference on Global Software Engineering, ICGSE 2010: 23 August 2010 through 26 August 2010; Princeton, NJ.* Edited by: Anonymous . 2010:45-54.

175. Jansen YJFM, Foets MME, De Bont AA: **The contribution of qualitative research to the development of tailor-made community-based interventions in primary care: A review.** *Eur J Public Health* 2010, **20**:220-226.

176. Jenkins E, Goldner EM: **Approaches to understanding and addressing treatment-resistant depression: a scoping review.** *Depress Res Treat* 2012, **2012**:469680.

177. Jepson R, Di Blasi Z, Wright K: *Scoping Review of the Effectiveness of Mental Health Services.* ; 2000.

178. Johnston R, Crooks VA, Snyder J, Kingsbury P: **What is known about the effects of medical tourism in destination and departure countries? A scoping review.** *Int J Equity Health* 2010, **9**:24.

179. Jones CA, Pohar S: **Health-Related Quality of Life After Total Joint Arthroplasty. A Scoping Review.** *Clin Geriatr Med* 2012, **28**:395.

180. Kademani BS, Surwase G, Sagar A, Kumar V: **Mapping of literature on Bose - Einstein Condensation.** *Malays J Libr Inf Sci* 2006, **11**:87-104.

181. Karsh J, Keystone EC, Haraoui B, Thorne JC, Pope JE, Bykerk VP, Maksymowych WP, Zummer M, Bensen WG, Kraishi MM, Canadian Rheumatology Research Consortium:

Canadian recommendations for clinical trials of pharmacologic interventions in rheumatoid arthritis: inclusion criteria and study design. *J Rheumatol* 2011, **38**:2095-2104.

182. Katz DL, Williams A-, Girard C, Goodman J, Comerford B, Behrman A, Bracken MB: **The evidence base for complementary and alternative medicine: Methods of evidence mapping with application to CAM.** *Altern Ther Health Med* 2003, **9**:22-30.

183. Kavanagh J, Stansfield C, Thomas J: *Incentives to improve smoking, physical activity, dietary and weight management behaviours: a scoping review of the research evidence.* London: EPPI-Centre, Social Science Research Unit, Institute of Education, University of London; 2009.

184. Kavanagh J, Trouton A, Oakley A, Harden A: *A scoping review of the evidence for incentive schemes to encourage positive behaviours in young people.* London: EPPI-Centre, Social Science Research Unit, Institute of Education, University of London; 2005.

185. Kendall S, Wilson P, Procter S, Brooks F, Bunn F, Gage H, McNeilly E: *The nursing contribution to chronic disease management: a whole systems approach.* Herts: Queen's Printer and Controller of HMSO; 2010.

186. Kirk SFL, Penney TL, McHugh T-F: **Characterizing the obesogenic environment: The state of the evidence with directions for future research.** *Obes Rev* 2010, **11**:109-117.

187. Kirst M, Zhang Y, Janice, Young A, Marshall A, O'Campo P, Ahmad F: **Referral to Health and Social Services for Intimate Partner Violence in Health Care Settings: A Realist Scoping Review.** *TRAUMA VIOLENCE ABUSE REV J* 2012, **13**:198-208.

188. Knowles E: *Workforce safety and hazards when attending emergency calls.* London: Department of Health Policy Research Programme

189. Koch S, Vimarlund V: **Critical advances in bridging personal health informatics and clinical informatics.** *Yearb Med Inform* 2012, **7**:48-55.

190. Koehlmoos T, Gazi R, Hossain S, Rashid M: *Social franchising evaluations: a scoping review.* London: EPPI-Centre, Social Science Research Unit, Institute of Education, University of London; 2011.

191. Kushki A, Chau T, Anagnostou E: **Handwriting Difficulties in Children with Autism Spectrum Disorders: A Scoping Review.** *J Autism Dev Disord* 2011, **41**:1-11.

192. Kusumo DS, Staples M, Zhu L, Zhang H and Jeffery R: Risks of off-the-shelf-based software acquisition and development: A systematic mapping study and a survey. *In: IET Seminar Digest:* Edited by: Anonymous . 2012:233-242.

193. Laguna MA, Crespo Y: **A systematic mapping study on software product line evolution: From legacy system reengineering to product line refactoring.** *Sci Comput Program* 2012, .

194. Lal S, Jarus T, Suto MJ: **A scoping review of the Photovoice method: Implications for occupational therapy research.** *Can J Occup Ther* 2012, **79**:181-190.

195. Leland N, E., Elliott S, J., OÂ¿Malley L, Murphy S, L.: **Occupational Therapy in Fall Prevention: Current Evidence and Future Directions.** *Am J Occup Ther* 2012, **66**:149-160.

196. Lemos J, Alves C, Duboc L and Rodrigues GN: A systematic mapping study on creativity in requirements engineering. *In: Proceedings of the ACM Symposium on Applied Computing:* Edited by: Anonymous . 2012:1083-1088.

197. Levac D, Wishart L, Missiuna C, Wright V: **The application of motor learning strategies within functionally based interventions for children with neuromotor conditions.** *Pediatr Phys Ther* 2009, **21**:345-355.

198. Levac D, Rivard L, Missiuna C: **Defining the active ingredients of interactive computer play interventions for children with neuromotor impairments: A scoping review.** *Res Dev Disabil* 2012, **33**:214-223.

199. Levasseur M, Carrier A: **Integrating health literacy into occupational therapy: findings from a scoping review.** *Scand J Occup Ther* 2012, **19**:305-314.

200. Liu P, Parker AG, Hetrick SE, Callahan P, de Silva S, Purcell R: **An evidence map of interventions across premorbid, ultra-high risk and first episode phases of psychosis.** *Schizophr Res* 2010, **123**:37-44.

201. Lopes Lobato L, Do Carmo Machado I, Da Mota Silveira Neto PA, Santana De Almeida E and Romero De Lemos Meira S: Risk management in software engineering: A scoping study. *In: IET Seminar Digest:* Edited by: Anonymous . 2012:243-252.

202. Lord P, Springate I, Atkinson M, Haines B, Morris M, O'Donnell L, Benefield P, Harper A, Sharp C: *Improving development outcomes for children through effective practice in integrating early years services; scoping review 1.* London: Centre for Excellence and Outcomes in Children and Young People's Services; 2008.

203. Lovell K, Bee P: **Optimising treatment resources for OCD: A review of the evidence base for technology-enhanced delivery.** *J Ment Heal* 2011, **20**:525-542.

204. Lysaght R, Cobigo V, Hamilton K: **Inclusion as a focus of employment-related research in intellectual disability from 2000 to 2010: a scoping review.** *Disabil Rehabil* 2012, **34**:1339-1350.

205. Macdonald JA, Edwards N, Davies B, Marck P, Guernsey JR: **Priority setting and policy advocacy by nursing associations: A scoping review and implications using a socio-ecological whole systems lens.** *Health Policy* 2012, **107**:31-43.

206. MacDougall A: **Is periodontal disease related to adverse pregnancy outcomes? A scoping review.** *Can J Dent Hyg* 2011, **45**:53-60.

207. MacEntee MI, Kazanjian A, Kozak JF, Hornby K, Thorne S, Kettratad-Pruksapong M: **A scoping review and research synthesis on financing and regulating oral care in long-term care facilities.** *Gerodontology* 2012, **29**:e41-52.

208. Magalhaes L, Carrasco C, Gastaldo D: **Undocumented migrants in Canada: A scope literature review on health, access to services, and working conditions.** *J Immigr Minor Health* 2010, **12**:132-151.

209. Maglyas A, Nikula U and Smolander K: What do we know about software product management? A systematic mapping study. *In: 2011 5th International Workshop on Software Product Management, IWSPM 2011 - Part of the 19th IEEE International Requirements Engineering Conference:* Edited by: Anonymous . 2011:26-35.

210. Mala A, Karkou V, Meekums B: **Dance/Movement Therapy (D/MT) for depression: A scoping review.** *Arts Psychother* 2012, **39**:287-295.

211. Manthorpe J, Livsey L: **European challenges in delivering social services in rural regions: A scoping review.** *Eur J Soc Work* 2009, **12**:5-24.

212. Manthorpe J, Martineau S: **Followers or leaders? What is the role for social care practitioners in annual health checks for adults with learning disabilities?** *J Intellect Disabil* 2010, **14**:53-66.

213. Manthorpe J, Martineau S: *Scoping review of the research and evidence base relating to advocacy services and older people's entry into care homes in England.* London: Social Care Workforce Research Unit, King's College London; 2009.

214. Manthorpe J, Martineau S, Moriarty J, Hussein S, Stevens M: **Support workers in social care in England: A scoping study.** *Health Soc Care Community* 2010, **18**:316-324.

215. Manthorpe J, Moriarty J, Cornes M: **Keeping it in the family? People with learning disabilities and families employing their own care and support workers: findings from a scoping review of the literature.** *J Intellect Disabil* 2011, **15**:195-207.

216. Marsella A: **Exploring the literature surrounding the transition into palliative care: a scoping review.** *Int J Palliat Nurs* 2009, **15**:186-189.

217. Marta FC, Correia AMR and Neves FT: Supporting KMS through cloud computing: A scoping review. *In: Proceedings of the 6th Iberian Conference on Information Systems and Technologies, CISTI 2011:* Edited by: Anonymous . 2011:

218. Martin K, Wilkin A, Morris M, O'Donnell L, Sharp C: *Improving the wellbeing of disabled children through early years interventions (age 0-18): scoping review 1.* London: Centre for Excellence and Outcomes in Children and Young People's Services; 2009.

219. Martin-Misener R, Valaitis R: *A Scoping Literature Review of Collaboration between Primary Care and Public Health: A Report to the Canadian Health Services Research Foundation.* Hamilton: McMaster University; 2008.

220. Martin-Misener R, Valaitis R, Wong ST, Macdonald M, Meagher-Stewart D, Kaczorowski J, O-Mara L, Savage R, Austin P, the Strengthening Primary Health Care through Public Health and Primary Care Collaborations Team: **A scoping literature review of collaboration between primary care and public health.** *Prim Health Care Res Dev* 2012, :1-20.

221. Masotti P, McColl MA, Green M: **Adverse events experienced by homecare patients: A scoping review of the literature.** *Int J Qual Health Care* 2010, **22**:115-125.

222. McColl MA, Shortt S, Godwin M, Smith K, Rowe K, O'Brien P, Donnelly C: **Models for Integrating Rehabilitation and Primary Care: A Scoping Study.** *Arch Phys Med Rehabil* 2009, **90**:1523-1531.

223. Medves J, Van Dijk J, Edgelow M, Saxe-Braithwaite M: *Scoping review of the Pre-Registration literature on curricula for interprofessional education. Report #1 of the Interprofessional Education Curricula Models for Health Care Providers in Ontario working group to the Interprofessional Care Strategic Implementation Committee.* Kingston: Queen's University; 2009.

224. Meredith A, Hussain Z, Griffiths MD: **Online gaming: A scoping study of massively multi-player online role playing games.** *Electron Commer Res* 2009, **9**:3-26.

225. Mitchell F, Lunt N, Shaw I: **Practitioner research in social work: A knowledge review.** *Evid Policy* 2010, **6**:7-31.

226. Mittmann N, Evans WK, Rocchi A, Longo CJ, Au HJ, Husereau D, Leighl NB, Isogai PK, Krahn MD, Peacock S, Marshall D, Coyle D, Taylor SC, Jacobs P, Oh PI: **Guidelines for health technologies: specific guidance for oncology products in Canada.** *Value Health* 2012, **15**:580-585.

227. Mitton C, Smith N, Peacock S, Evoy B, Abelson J: **Public participation in health care priority setting: A scoping review.** *Health Policy* 2009, **91**:219-228.

228. Mohd Fauzi SS, Bannerman PL and Staples M: Software Configuration Management in Global Software Development: A systematic map. *In: 17th Asia Pacific Software Engineering Conference: Software for Improving Quality of Life, APSEC 2010: 30 November 2010 through 3 December 2010; Sydney, NSW.* Edited by: Anonymous . 2010:404-413.

229. Mohebzada JG, Ruhe G and Eberlein A: Systematic mapping of recommendation systems for requirements engineering. *In: 2012 International Conference on Software and System Process, ICSSP 2012 - Proceedings:* Edited by: Anonymous . 2012:200-209.

230. Mombelli A, Decaillet F: **The characteristics of biofilms in peri-implant disease.** *J Clin Periodontol* 2011, **38 Suppl 11**:203-213.

231. Morelli LB and Nakagawa EY: A panorama of software architectures in game development. *In: SEKE 2011 - Proceedings of the 23rd International Conference on Software Engineering and Knowledge Engineering:* Edited by: Anonymous . 2011:752-757.

232. Moreno-Casbas T: **Nursing Research In Europe: A Scoping Report.** *European Commission (ERA-CARE), 1-133.Madrid Spain, European Union 6th Framework Programme Instituto de Salud* 2005, :1-133.

233. Moriarty J, Manthorpe J, Stevens M, Hussein S: **Making the transition: Comparing research on newly qualified social workers with other professions.** *Br J Soc Work* 2011, **41**:1340-1356.

234. Mountain GA: **Self-management for people with early dementia: an exploration of concepts and supporting evidence.** *Dementia* 2006, **5**:429-446.

235. Munro ER: *The protection of children online: a brief scoping review to identify vulnerable groups.* London: Childhood Wellbeing Research Centre; 2011.

236. Murray SF, Pearson SC: **Maternity referral systems in developing countries: Current knowledge and future research needs.** *Soc Sci Med* 2006, **62**:2205-2215.

237. Murugesupillai E, Mohabbati B and Gaševic D: A preliminary mapping study of approaches bridging software product lines and service-oriented architectures. *In: ACM International Conference Proceeding Series:* Edited by: Anonymous . 2011:

238. Naylor C, Appleby J: *Environmentally sustainable health and social care: Scoping Review.* London: NIHR Service Delivery and Organisation Programme; 2012.

239. Neal RD: **Do diagnostic delays in cancer matter.** *Br J Cancer* 2009, **101**:S9-S12.

240. Neves FT, Rosa VN, Correia AMR and Neto MDC: Knowledge creation and sharing in software development teams using Agile methodologies: Key insights affecting their adoption. *In: Proceedings of the 6th Iberian Conference on Information Systems and Technologies, CISTI 2011:* Edited by: Anonymous . 2011:

241. Neves J, Lavis JN, Ranson MK: **A scoping review about conference objectives and evaluative practices: how do we get more out of them?** *Health Res Policy Syst* 2012, **10**:26.

242. Newbigging K, Bola M, Shah A: *Scoping exercise with black and minority ethnic groups on perceptions of mental wellbeing in Scotland.* Edinburgh: NHS Health Scotland; 2008.

243. Nguyen L, Cobban S, J., Keenan L: **Caffeine as an adjuvant to common over the counter analgesics for postoperative dental pain: A scoping review.** *CAN J DENT HYG* 2012, **46**:57-62.

244. Nicolson P, Beverly C, Booth A, Burr J, Collins K, Cooper C, O'Cathain A, Powell J: *Eliciting Users' Views of the Processes of Health Care: A Scoping Exercise.* University of Sheffield; 2000.

245. Njelesani J, Couto S, Cameron D: **Disability and rehabilitation in Tanzania: a review of the literature.** *Disabil Rehabil* 2011, **33**:2196-2207.

246. Nolte E, Roland M, Guthrie S, Brereton L: *Preventing emergency readmissions to hospital: A scoping review.* Cambridge: RAND Europe; 2012.

247. Northway R, Davies R, Jenkins R, Mansell I, Fairbairn G: *Abuse of people with learning disabilities: an examination of policy, practice and educational implications in Wales.* Pontypridd: School of Care Sciences, University of Glamorgan; 2004.

248. O'Brien K, Bone G, Zack E, Solomon P: **HIV and rehabilitation: Development of a conceptual framework for curriculum planning.** *Int J Rehabil Res* 2008, **31**:189-197.

249. O'Brien K, Wilkins A, Zack E, Solomon P: **Scoping the field: Identifying key research priorities in HIV and rehabilitation.** *AIDS Behav* 2010, **14**:448-458.

250. O'Cathain A: *Patient involvement in planning of emergency care.* London: Department of Health Policy Research Programme; 2010.

251. O'Cathain A: *Equality of access.* London: Department of Health Policy Research Programme; 2010.

252. Ogilvie I, Khoury H, Goetghebeur M, M., C, Giaquinto C: **Burden of community-acquired and nosocomial rotavirus gastroenteritis in the pediatric population of Western Europe: a scoping review.** *BMC Infect Dis* 2012, **12**:62-62.

253. O'Keeffe C, Mason M: *Post-traumatic stress disorder (PTSD) in ambulance staff.* London: Department of Health Policy Research Programme

254. O'Malley L, Croucher K: **Housing and dementia care - A scoping review of the literature.** *Health Soc Care Community* 2005, **13**:570-577.

255. O'Malley L, Croucher K: **Supported housing services for people with mental health problems: A scoping study.** *Hous Stud* 2005, **20**:831-845.

256. O'Reilly G, Cameron PA, Joshipura M: **Global trauma registry mapping: A scoping review.** *Injury* 2012, **43**:1148-1153.

257. Pagliari C, Sloan D, Gregor P, Sullivan F, Detmer D, Kahan JP, Oortwijn W, MacGillivray S: **What is eHealth (4): A scoping exercise to map the field.** *J Med Internet Res* 2005, **7**.

258. Parke B, Beaith A, Slater L, Clarke AM: **Contextual factors influencing success or failure of emergency department interventions for cognitively impaired older people: a scoping and integrative review.** *J Adv Nurs* 2011, **67**:1426-1448.

259. Parker G, Arksey H, Harden M: *Meta-review of International Evidence on Interventions to Support Carers.* York: Social Policy Research Unit, University of York; 2010.

260. Paterson BL, Brewer J, Stamler LL: **Engagement of Parents in On-line Social Support Interventions.** *J Pediatr Nurs* 2012, .

261. Pattison S, Harris B: **Counselling children and young people: a review of the evidence for its effectiveness.** *CPR* 2006, **6**:233-237.

262. Peat M, Entwistle V, Hall J, Birks Y, Golder S: **Scoping review and approach to appraisal of interventions intended to involve patients in patient safety.** *J Health Serv Res Policy* 2010, **15**:17-25.

263. Petersen K: **Measuring and predicting software productivity: A systematic map and review.** *Inf Software Technol* 2011, **53**:317-343.

264. Philips Z, Ginnelly L, Sculpher M, Claxton K, Golder S, Riemsma R, Woolacott N, Glanville J: **Review of guidelines for good practice in decision-analytic modelling in health technology assessment.** *Health Technol Assess* 2004, **8**:47-51.

265. PHIRN Scoping Review Committee: *Scoping Review of the Population Health Equity and Intervention Literature in Ontario.* Ottawa: Population Health Improvement Research Network; 2012.

266. Pita C, Pierce GJ, Theodossiou I, Macpherson K: **An overview of commercial fishers' attitudes towards marine protected areas.** *Hydrobiologia* 2011, **670**:289-306.

267. Pitt M, Stahl-Timmins W, Anderson R, Stein K: **Using information graphics in health technology assessment: Toward a structured approach.** *Int J Technol Assess Health Care* 2009, **25**:555-563.

268. Plow MA, Finlayson M, Rezac M: **A Scoping Review of Self-Management Interventions for Adults With Multiple Sclerosis.** *PM R* 2011, **3**:251-262.

269. Portillo-Rodríguez J, Vizcaíno A, Piattini M, Beecham S: **Tools used in Global Software Engineering: A systematic mapping review.** *Inf Software Technol* 2012, **54**:663-685.

270. Prodinger B, Magalhães L: **Advancing knowledge in work-related rehabilitation - Review of research published in the journal of WORK.** *Work* 2010, **35**:301-318.

271. Qadir MM and Usman M: Software engineering curriculum: A systematic mapping study. *In: 2011 5th Malaysian Conference in Software Engineering, MySEC 2011:* Edited by: Anonymous . 2011:269-274.

272. Raghoonandan P, Cobban S, J., Compton S, M.: **A scoping review of the use of fluoride varnish in elderly people living in long term care facilities.** *CAN J DENT HYG* 2011, **45**:217-222.

273. Ravenek MJ, Bryson-Campbell MM, Shaw L, Hughes ID: **Perspectives on prevention, assessment, and rehabilitation of low back pain in WORK.** *Work* 2010, **35**:269-282.

274. Redwood-Campbell L, Pakes B, Rouleau K, MacDonald CJ, Arya N, Purkey E, Schultz K, Dhatt R, Wilson B, Hadi A, Pottie K: **Developing a curriculum framework for global health in family medicine: emerging principles, competencies, and educational approaches.** *BMC Med Educ* 2011, **11**:46-46.

275. Reeves S, Goldman J, Gilbert J, Tepper J, Silver I, Suter E, Zwarenstein M: **A scoping review to improve conceptual clarity of interprofessional interventions.** *J Interprof Care* 2011, **25**:167-174.

276. Richardson R, Richards DA, Barkham M: **Self-help books for people with depression: A scoping review.** *J Ment Health* 2008, **17**:543-552.

277. Ridde V, Morestin F: **A scoping review of the literature on the abolition of user fees in health care services in Africa.** *Health Policy Plann* 2011, **26**:1-11.

278. Ridde V, Robert E, Meesen B: *Les pressions exercées par l'abolition du paiement des soins sur les systèmes de santé.* Geneva: World Health Organization; 2010.

279. Rodgers M, Asaria M, Walker S, McMillan D, Lucock M, Harden M, Palmer S, Eastwood A: **The clinical effectiveness and cost-effectiveness of low-intensity psychological interventions for the secondary prevention of relapse after depression: a systematic review.** *Health Technol Assess* 2012, **16**:1-130.

280. Roland M, McDonald R, Sibbald B: *Outpatient Services and Primary Care: A scoping review of research into strategies for improving outpatient effectiveness and efficiency.* Southampton: National Co-ordinating Centre for the NIHR SDO (NCCSDO); 2006.

281. Sanmartin C, Murphy K, Choptain N, Conner-Spady B, McLaren L, Bohm E, Dunbar MJ, Sanmugasunderam S, De Coster C, McGurran J, Lorenzetti DL, Noseworthy T: **Appropriateness of healthcare interventions: Concepts and scoping of the published literature.** *Int J Technol Assess Health Care* 2008, **24**:342-349.

282. Saparova D: **Motivating, Influencing, and persuading patients through personal health records: a scoping review.** *Perspect Health Inf Manag* 2012, **9**:1f.

283. Saraiva J, Barreiros E, Almeida A, et al: Aspect-oriented software maintenance metrics: A systematic mapping study. *In: Proceedings of the 16th International Conference on Evaluation & Assessment in Software Engineering: 14-15 May; Ciudad Real, Spain.* Edited by: Baldassarre MT, Genero M, Mendes E, Piattini M. United Kingdom: The Institution of Engineering and Technology; 2012:253-262.

284. Sawka AM, Ismaila N, Cranney A, Thabane L, Kastner M, Gafni A, Woodhouse LJ, Crilly R, Cheung AM, Adachi JD, Josse RG, Papaioannou A: **A scoping review of strategies for the prevention of hip fracture in elderly nursing home residents.** *PLoS ONE* 2010, **5**:e9515.

285. Schaink AK, Kuluski K, Lyons RF, Fortin M, Jadad AR, Upshur R, Wodchis WP: **A scoping review and thematic classifi cation of patient complexity: offering a unifying framework.** *Journal of Comorbidity* 2012, **2**:1-9.

286. Scott NA, Moga C, Harstall C: **Managing low back pain in the primary care setting: the know-do gap.** *Pain Res Manag* 2010, **15**:392-400.

287. Scott RE, Saunders C, Palacios M, Nguyen DTK and Ali S: Healthy e-Health? think 'Environmental e-Health'! *In: 2010 Conference on Global Telehealth, GT2010: 10 November 2010 through 12 November 2010; Fremantle, Perth, WA.* Edited by: Anonymous . 2010:132-138.

288. Seekins T, Shunkamolah W, Bertsche M, Cowart C, Summers JA, Reichard A, White G: **A systematic scoping review of measures of participation in disability and rehabilitation research: A preliminary report of findings.** *Disabil Health J* 2012, .

289. Shachak A, Barnsley J, Tu K, Jadad AR, Lemieux-Charles L: **Understanding end-user support for health information technology: A theoretical framework.** *Informatics Prim Care* 2012, **19**:169-172.

290. Shankardass K, Solar O, Murphy K, Greaves L, O'Campo P: **A scoping review of intersectoral action for health equity involving governments.** *Int J Public Health* 2012, **57**:25-33.

291. Siraj-Blatchford I, Siraj-Blatchford J: *Improving children's attainment through a better quality of family-based support for early learning.* London: Centre for Excellence and Outcomes in Children and Young People's Services; 2009.

292. Snyder J, Crooks VA, Johnston R, Kingsbury P: **What do we know about Canadian involvement in medical tourism?: a scoping review.** *Open Med* 2011, **5**:e139-48.

293. Spilsbury K, Hewitt C, Stirk L, Bowman C: **The relationship between nurse staffing and quality of care in nursing homes: A systematic review.** *Int J Nurs Stud* 2011, **48**:732-750.

294. Stalker K, Mcarthur K: **Child abuse, child protection and disabled children: A review of recent research.** *Child Abuse Rev* 2012, **21**:24-40.

295. Steinmacher I, Chaves AP, Gerosa MA: **Awareness Support in Distributed Software Development: A Systematic Review and Mapping of the Literature.** *Comput Supported Coop Work CSCW Int J* 2012, :1-46.

296. Stelfox HT, Bobranska-Artiuch B, Nathens A, Straus SE: **Quality indicators for evaluating trauma care a scoping review.** *Arch Surg* 2010, **145**:286-295.

297. Sunley R, Locke R: **Exploring UK secondary teachers' professional values: An overview of the literature since 2000.** *Educ Res* 2010, **52**:409-425.

298. Surette S, Vanderjagt L, Vohra S: **Surveys of complementary and alternative medicine usage: A scoping study of the paediatric literature.** *Complement Ther Med* .

299. Symonds JE, Hagell A: **Adolescents and the organisation of their school time: A review of changes over recent decades in England.** *Educ Rev* 2011, **63**:291-312.

300. Templeton L, Zohhadi S, Galvani S, Velleman R: *"Looking Beyond Risk" Parental Substance Misuse: Scoping Study.* Edinburgh: Scottish Executive; 2006.

301. Terstappen V, Hanson L, McLaughlin D: **Gender, health, labor, and inequities: a review of the fair and alternative trade literature.** *Agric Hum Values* 2012, :1-19.

302. To-Miles F, Shaw L: **Knowledge transfer with children and adolescents in promoting comfort, health, and safety in technology use: Strategies and opportunities.** *Work* 2012, .

303. Toohey AM, Rock MJ: **Unleashing their potential: A critical realist scoping review of the influence of dogs on physical activity for dog-owners and non-owners.** *International Journal of Behavioral Nutrition and Physical Activity* 2011, :46.

304. Traynor M, Davis K, Drennan V, Goodman C, Humphrey C, Locke R, Mark A, Murray SF, Banning M, Peacock R: *The Contribution of Nurse, Midwife and Health Visitor Entrepreneurs to Patient Choice: A scoping exercise: A report to the National Co-ordinating Centre for NHS Service Delivery and Organisation.* London: National Co-ordinating Centre for NHS Service Delivery and Organisation; 2007.

305. Trivedi D, Brooks F, Bunn F, Graham M: **Early fatherhood: A mapping of the evidence base relating to pregnancy prevention and parenting support.** *Health Educ Res* 2009, **24**:999-1028.

306. Turner J: *Alternatives to ambulance response of transportation to A&E.* London: Department of Health Policy Research Programme; 2010.

307. Turner J: *Epidemiology and understanding demand for 999 ambulance services.* London: Department of Health Policy Research Programme; 2010.

308. Tusevljak N, Rajic A, Waddell L, Dutil L, Cernicchiaro N, Greig J, Wilhelm BJ, Wilkins W, Totton S, Uhland FC, Avery B, McEwen SA: **Prevalence of zoonotic bacteria in wild and farmed aquatic species and seafood: a scoping study, systematic review, and meta-analysis of published research.** *Foodborne Pathog Dis* 2012, **9**:487-497.

309. University of Sheffield Medical Care Research Unit: *Control room assessment.* London: Department of Health Policy Research Programme; 2010.

310. University of Sheffield Medical Care Research Unit: *Near patient assessment.* London: Department of Health Policy Research Programme; 2010.

311. Van Dijk J, Medves J, Edgelow M, Saxe-Braithwaite M: *Scoping review of Post-Registration (Continuing Education and Post-Graduate) literature on curricula for interprofessional education. Report #2 of the Interprofessional Education Curricula Models for Health Care Providers in Ontario working group to the Interprofessional Care Strategic Implementation Committee.* Kingston: Queen's University; 2009.

312. Van Mossel C, Leitz L, Scott S, Daudt H, Dennis D, Watson H, Alford M, Mitchell A, Payeur N, Cosby C, Levi-Milne R, Purkis ME: **Information needs across the colorectal cancer care continuum: scoping the literature.** *EUR J CANCER CARE* 2012, **21**:296-320.

313. Victoor A, Delnoij DM, Friele RD, Rademakers JJ: **Determinants of patient choice of healthcare providers: a scoping review.** *BMC Health Serv Res* 2012, **12**:272.

314. Vis SA, Strandbu A, Holtan A, Thomas N: **Participation and health - a research review of child participation in planning and decision-making.** *Child Fam Soc Work* 2011, **16**:325-335.

315. Vissandjee B, Hyman I, Spitzer DL, Apale A, Kamrun N: **Integration, clarification, substantiation: Sex, gender, ethnicity and migration as social determinants of women's health.** *J Int Women's Stud* 2007, **8**:32-48.

316. Wallace LM, Koutantji M, Spurgeon P, Vincent C, Benn J, Earll L: *Reporting Systems: a scoping study of methods of providing feedback within an organization - Report to the Department of Health Patient Safety Research Programme.* United Kingdom: Department of Health Patient Safety Research Programme; 2006.

317. Watson R, Parr J, R., Joyce C, May C, S.: **Models of transitional care for young people with complex health needs: a scoping review.** *Child Care Health Dev* 2011, **37**:780-791.

318. Weeks LC, Strudsholm T: **A scoping review of research on complementary and alternative medicine (CAM) and the mass media: Looking back, moving forward.** *BMC Complement Altern Med* 2008, **8**.

319. Wendler R: **The maturity of maturity model research: A systematic mapping study.** *Inf Software Technol* 2012, **54**:1317-1339.

320. While A, Forbes A, Ullman R, Murgatroyd B: *The contribution of nurses, midwives and health visitors to child health and child health services: a scoping review.* Southampton: National Co-ordinating Centre for NHS Service Delivery and Organisation; 2005.

321. White DE, Straus SE, Stelfox HT, Holroyd-Leduc JM, Bell CM, Jackson K, Norris JM, Flemons WW, Moffatt ME, Forster AJ: **What is the value and impact of quality and safety teams? A scoping review.** *Implement Sci* 2011, **6**:97-97.

322. Wilhelm BJ, Rajic A, Greig J, Waddell L, Trottier G, Houde A, Harris J, Borden LN, Price C: **A systematic review/meta-analysis of primary research investigating swine, pork or pork products as a source of zoonotic hepatitis E virus.** *Epidemiol Infect* 2011, :1-18.

323. Williams AP, Deber R, Lum JM, Montgomery R, Peckham A, Kuluski K, Watkins J, Morton-Chang F, Williams A, Ying A, Zhu L: *Mapping the state of the art: Integrating care for vulnerable older populations.* Toronto, Ontario: Canadian Research Network for Care in the Community; 2009.

324. Williams R, M., Bambara J, Turner A, P.: **A Scoping Study of One-to-One Peer Mentorship Interventions and Recommendations for Application With Veterans With Postdeployment Syndrome.** *J Head Trauma Rehabil* 2012, **27**:261-273.

325. Williams V, Marriott A, Townsley R: *Shaping our future: a scoping and consultation exercise to establish research priorities in learning disabilities for the next ten years.* London: Queen's Printer and Controller of HMSO; 2008.

326. Williams-Brennan L, Gastaldo D, Cole DC, Paszat L: **Social determinants of health associated with cervical cancer screening among women living in developing countries: a scoping review.** *Arch Gynecol Obstet* 2012, .

327. Wilson MG, Dickie M, Cooper CL, Carvalhal A, Bacon J, Rourke SB: **Treatment, care and support for people co-infected with HIV and hepatitis C: a scoping review.** *Open Med* 2009, **3**:e184-95.

328. Wilson PM, Petticrew M, Calnan MW, Nazareth I: **Disseminating research findings: What should researchers do? A systematic scoping review of conceptual frameworks.** *Implement Sci* 2010, **5**.

329. Wilson R, Goodacre S: *Information and performance measurement.* London: Department of Health Policy Research Programme; 2010.

330. Wong ST, MacLeod MLP, Farrally V: *Health Human Resource: Scoping Literature Review and Synthesis.* Ottawa: Michael Smith Foundation for Health Research; 2009.

331. Worthington C, O'Brien K, Zack E, Mckee E, Oliver B: **Enhancing labour force participation for people living with HIV: a multi-perspective summary of the research evidence.** *AIDS Behav* 2011, .

332. Wysocki A, Butler M, Shamliyan T, Kane RL: 2011, .

333. Young R, Weir H, Buchan J: *Health Professional Mobility in Europe and the UK: A Scoping Study of Issues and Evidence.* Great Britain: Queen’s Printer and Controller of HMSO; 2010.

334. Younger P: **Internet-based information-seeking behaviour amongst doctors and nurses: a short review of the literature.** *Health Info Libr J* 2010, **27**:2-10.

335. Ziegler L, Hook J, Stark D, Neilly L, Hodges L, Walker J, Sharpe M: *Systematic scope and collation of research evidence regarding intervention. Technical Report.* United Kingdom: University of Leeds and University of Edinburgh; 2009.
